# Targeting HIV Reservoir in Infected CD4 T Cells by Dual-Affinity Re-targeting Molecules (DARTs) that Bind HIV Envelope and Recruit Cytotoxic T Cells

**DOI:** 10.1371/journal.ppat.1005233

**Published:** 2015-11-05

**Authors:** Derek D. Sloan, Chia-Ying Kao Lam, Alivelu Irrinki, Liqin Liu, Angela Tsai, Craig S. Pace, Jasmine Kaur, Jeffrey P. Murry, Mini Balakrishnan, Paul A. Moore, Syd Johnson, Jeffrey L. Nordstrom, Tomas Cihlar, Scott Koenig

**Affiliations:** 1 Gilead Sciences, Foster City, California, United States of America; 2 MacroGenics, Inc., Rockville, Maryland, United States of America; John Hopkins University, UNITED STATES

## Abstract

HIV reservoirs and production of viral antigens are not eliminated in chronically infected participants treated with combination antiretroviral therapy (cART). Novel therapeutic strategies aiming at viral reservoir elimination are needed to address chronic immune dysfunction and non-AIDS morbidities that exist despite effective cART. The HIV envelope protein (Env) is emerging as a highly specific viral target for therapeutic elimination of the persistent HIV-infected reservoirs via antibody-mediated cell killing. Dual-Affinity Re-Targeting (DART) molecules exhibit a distinct mechanism of action via binding the cell surface target antigen and simultaneously engaging CD3 on cytotoxic T lymphocytes (CTLs). We designed and evaluated Env-specific DARTs (HIVxCD3 DARTs) derived from known antibodies recognizing diverse Env epitopes with or without broadly neutralizing activity. HIVxCD3 DARTs derived from PGT121, PGT145, A32, and 7B2, but not VRC01 or 10E8 antibodies, mediated potent CTL-dependent killing of quiescent primary CD4 T cells infected with diverse HIV isolates. Similar killing activity was also observed with DARTs structurally modified for in vivo half-life extension. In an ex vivo model using cells isolated from HIV-infected participants on cART, combinations of the most potent HIVxCD3 DARTs reduced HIV expression both in quiescent and activated peripheral blood mononuclear cell cultures isolated from HIV-infected participants on suppressive cART. Importantly, HIVxCD3 DARTs did not induce cell-to-cell virus spread in resting or activated CD4 T cell cultures. Collectively, these results provide support for further development of HIVxCD3 DARTs as a promising therapeutic strategy for targeting HIV reservoirs.

## Author Summary

Current HIV therapies prevent AIDS by dramatically reducing, but not eliminating, HIV infection. A reservoir of HIV-infected cells persists during long-term antiviral therapy, and individuals are at increased risk to develop non-AIDS illnesses, e.g., accelerated heart, bone, or kidney disease. Novel strategies are thus needed to safely kill HIV-infected cells and reduce or eliminate the HIV reservoir. An emerging strategy to kill HIV-infected cells involves antibodies (Abs) that bind the HIV envelope protein (Env). Env can distinguish HIV-infected cells from uninfected cells, and some Env-specific Abs can kill HIV-infected cells by recruiting immune cells, e.g., NK cells and macrophages. Here, we developed a strategy to kill HIV-infected cells that is complementary to Env-specific Abs. We designed and evaluated Dual-Affinity Re-Targeting (DART) molecules that incorporate Env-binding specificities with a CD3-binding specificity to recruit and activate cytotoxic T cells. We report that HIVxCD3 DARTs potently and selectively kill HIV-infected cells. Furthermore, HIV DARTs perturb resting and activated viral reservoirs in cells isolated from individuals on antiviral therapy. This novel strategy may be an important element of future antiviral therapies that target the HIV reservoir.

## Introduction

Advanced regimens of combination antiretroviral therapy (cART) prevent AIDS and suppress HIV replication to nearly undetectable levels in over 90% of treatment-naïve participants [1–3]. However, in nearly all cases, cART interruption results in resumption of viral replication [4,5], which indicates that current cART is not sufficient to eliminate the HIV reservoir and cure persistent infection. The ability of HIV to establish latency in a subset of infected CD4 T cells limits the ability of cART to reduce the reservoir [6]. Latency is characterized by the presence of integrated but transcriptionally silent proviral HIV DNA, which makes the infected cells invisible to the immune system and resistant to innate antiviral defenses [6,7].

Proviral DNA has been detected in multiple immune cell subsets that are permissive to HIV infection, but the best characterized reservoir exists in long-lived resting memory CD4 T cells [7,8]. The rare pool of latently infected memory CD4 T cells capable of producing infectious virus upon activation is believed to be maintained by homeostatic proliferation of memory T cells and/or intermittent antigen-driven clonal expansion [9]. Low levels of HIV replication confined to lymphatic tissues and undetectable in the periphery may also contribute the HIV reservoir [10,11]. Additionally, there is evidence that persistently infected cells capable of expressing low but detectable levels of HIV protein exist [12,13]. Herein, the HIV reservoir is defined to encompass: latently infected cells that are transcriptionally silent, persistently infected cells that express HIV protein basally, and cells that can be activated to increase expression of HIV protein. The extended decay rate of HIV reservoirs in peripheral blood lymphocytes indicates that life-long treatment with current cART regimens is unlikely to cure HIV infection [7].

Despite the success of cART in reducing viremia, HIV can be detected in participants on suppressive cART using sensitive single-copy assays [14]. Antiviral drugs do not prevent viral antigen expression in HIV-infected cells, which may contribute to chronic immune activation and inflammation in participants on cART [15–17]. Together, persistent HIV infection and associated immune dysfunction increase the long-term risk for non-AIDS morbidities including accelerated cardiovascular disease, liver and renal disease, non-AIDS-associated cancers, and neurocognitive impairment [18–20]. Thus, therapeutic interventions are needed that could substantially reduce or eliminate the HIV reservoirs or, alternatively, lead to host-mediated control of HIV without cART [10]. One proposed strategy is to combine pharmacologic activation of latent HIV expression with immune-mediated elimination of infected cells. Various classes of latency reversal agents such as HDAC inhibitors or TLR7 agonists have demonstrated the ability to activate the quiescent reservoir and increase viral gene expression ex vivo and/or in vivo [21,22]. HIV envelope protein (Env) is an attractive target for immune-mediated killing of infected cells because it is unique to the virus. Potent broadly neutralizing anti-Env IgG antibodies (bNAbs) with preserved Fc-dependent effector function have provided preliminary in vivo evidence for reservoir reduction [23,24]. Bi-specific antibodies that combine an anti-Env arm with an anti-CD3 arm to recruit CTLs are an alternative strategy to IgG-mediated killing. This strategy could recruit CTLs with any T cell receptor specificity to selectively kill HIV-infected cells.

Bi-specific antibodies have demonstrated the ability to potently kill low-frequency target cells in humans, e.g., residual disease in B-cell lymphoma [25,26]. Several bi-specific platforms are in various stages of clinical testing, primarily for oncology-based therapeutic applications. Bi-specific T-cell engagers (BiTEs) represent one of the most advanced bi-specific antibody platforms, as blinatumomab (CD19xCD3 BiTE) was recently approved for the treatment of acute lymphoblastic leukemia [27]. An alternative platform, termed Dual-Affinity Re-Targeting (DART) currently has several constructs in clinical development for oncology indications: MGD006, a CD123xCD3 DART, is being evaluated in a Phase 1 trial in patients with refractory acute myeloid leukemia [28] (NCT02152956); MGD007, a gpA33xCD3 DART in MP3 format for enhanced pharmacokinetic (PK) properties, is being evaluated in a Phase 1 trial in patients with colorectal cancer (NCT02248805); MGD011, a CD19xCD3 DART in MP3 format, is being evaluated in a Phase 1 trial in patients with B-cell hematological malignancies (NCT02454270). DART molecules also are being pursued to attenuate autoimmune disorders: MGD010, a CD32BxCD79B DART in MP3 format, is designed to simultaneously bind both targets on individual B cells and inhibit their activation; it is being tested in a Phase 1 trial in normal volunteers (NCT02376036).

To examine the potential of employing the DART platform to treat participants with HIV, we describe here the design and characterization of a series of HIVxCD3 DARTs derived from diverse broadly reactive anti-Env monoclonal antibodies that are non-neutralizing or neutralizing. The DART constructs induced a potent and specific CD8 T cell-dependent elimination of primary resting CD4 T cells infected with multiple HIV isolates in vitro. In addition, HIVxCD3 DARTs were capable of reducing the level of virion production ex vivo in cells isolated from infected participants on suppressive antiretroviral therapy. Together, the generated data support further development of these bi-specific T-cell redirecting molecules for targeting the HIV reservoirs in cART-treated participants.

## Results

### Design and Production of HIVxCD3 DARTs

**Fig 1:**
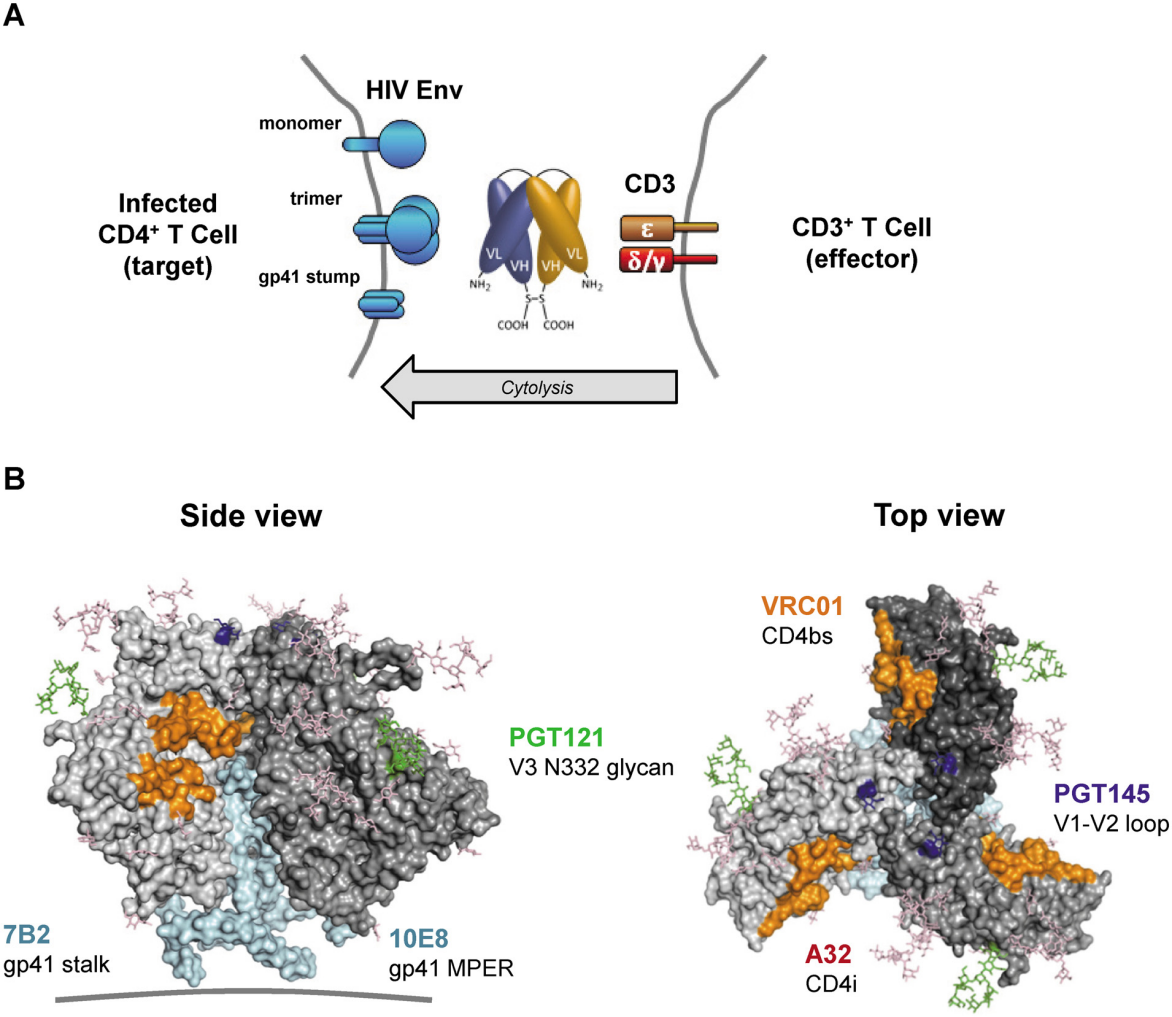
HIVxCD3 DARTs retarget cytolytic CD3^+^ T-cells to Env-expressing HIV-infected CD4^+^ T-cells. (A) Mechanism of cytolysis. The CD3 arm (orange) of the bi-specific DART binds to CD3 at the surface of CD3^+^ T-cells and the HIV arm (blue) binds to HIV Env at the surface of HIV-infected CD4^+^ T-cells. Cell surface Env may be in the form of functional mature trimers or nonfunctional variant forms such as cleaved or uncleaved gp160 monomers or gp41 stumps [43]. DART-mediated engagement of target and effector cells results in activation of effector cell cytolytic responses and target cell killing. (B) Variety of Env epitopes targeted by HIVxCD3 DARTs. Locations on the mature HIV-1 Env trimer surface of epitopes recognized by the anti-Env Abs used as sources of the HIV binding arms of DARTs are shown. Broadly neutralizing Abs PGT121, PGT145, VRC01 and 10E8 target epitopes located in the V3 glycan (N332; green), V2 glycan (N160K, blue), CD4 binding site (CD4bs, orange) and gp41 MPER (cyan), respectively, that are preferentially expressed on functional Env trimers, whereas non-neutralizing Abs A32 and 7B2 target epitopes located in CD4-induced sites (CD4i conformation epitopes are not visible in the depicted pre-CD4 binding Env structure) and in the gp41 stalk (cyan), respectively, that are preferentially expressed on nonfunctional forms of Env. The depicted structure of Env trimer is derived from pdb 4NCO.

A series of bi-specific antibody constructs that bind simultaneously to HIV Env and human CD3 receptor were generated using the basic DART platform (Fig 1A) [29,30]. To maximize the breadth of Env recognition across multiple HIV isolates, complementarity determining regions (CDRs) from four bNAbs (PGT121, PGT145, 10E8, VRC01) [31–33] were incorporated into the Env-recognizing arm of HIVxCD3 DARTs. These bNAb-derived DARTs were compared with ones with HIV Env arms derived from two non-neutralizing antibodies (A32, 7B2) that bind broadly conserved residues in Env and efficiently induce antibody dependent cell-mediated cytotoxicity (ADCC) [34–41]. Each of the six designed bi-specific Abs recognizes a distinct epitope on the surface of HIV Env protein (Fig 1B). CDRs derived from palivizumab [42], an antibody recognizing the fusion protein of respiratory syncytial virus (RSV), were used to construct a negative control DART (RSVxCD3) that does not bind to HIV Env. The CD3-recognizing arm, which was identical in all DARTs, was derived from hXR32 [28], a humanized mouse anti-human CD3ε antibody, which cross-reacts with nonhuman primate CD3ε. The HIVxCD3 DARTs in basic format were produced by expression in stably transfected CHO cells and purified. The formation of properly assembled molecules was confirmed by reducing and non-reducing SDS-PAGE and analytical SEC; the average purity of the assembled HIVxCD3 DART molecules was 95%.

**Fig 2:**
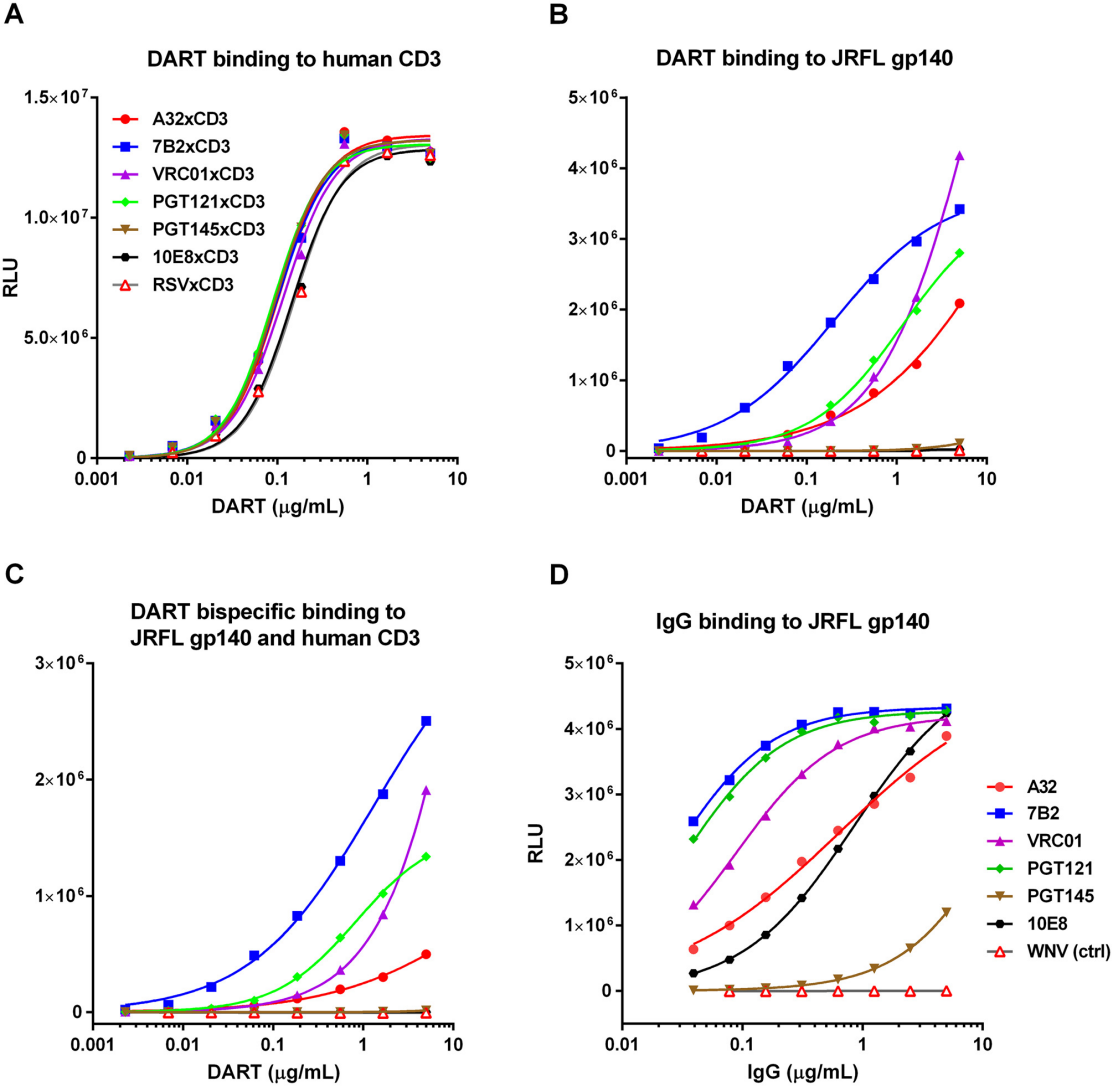
Antigen binding properties of HIVxCD3 DARTs and parental anti-HIV Env IgGs. Binding of DARTs to (A) soluble recombinant human CD3 protein, to (B) JRFL gp140 protein, or to (C) both soluble recombinant human CD3 and JRFL gp140 proteins. Binding of IgGs with CDRs exactly matching those utilized in the HIV arms of HIVxCD3 DARTs to (D) JRFL gp140 protein was measured by ELISA, as described in Materials and Methods. WNV IgG (negative control) is an antibody to the envelope protein of West Nile virus.

DART binding via the CD3 arm to soluble recombinant human CD3 receptor was equivalently efficient for all HIVxCD3 DARTs as well as for the RSVxCD3 control (Fig 2A). DART binding via the HIV Env arm to soluble recombinant HIV JRFL Env (gp140) monomer ranged from efficient (7B2, VRC01, PGT121) to less efficient (A32) to undetectable (PGT145, 10E8) (Fig 2B). These patterns were recapitulated in the bi-specific binding assay, which measures simultaneous binding to JRFL gp140 and human CD3 (Fig 2C). Weaker gp140 binding by the A32 arm is consistent with the CD4-inducible nature of its epitope [34,36], as binding was assessed in the absence of CD4. Lack of gp140 monomer binding by the PGT145 arm is consistent with the quaternary nature of its epitope (V1-V2 loop), which exists only in mature Env trimers [32]. Lack of gp140 monomer binding by the 10E8 arm may be due to the dependence of its epitope, located in the membrane-proximal external region (MPER), on the fusion-intermediate conformational state of gp41 [44]. In general, the binding of the HIVxCD3 DARTs to JRFL gp140 mimicked that of the corresponding parental IgGs (Fig 2D). All 6 HIVxCD3 DARTs exhibited binding to CM244 and/or 92Th023 Env (gp140) presented on the surface of Env-transfected HEK293 cells (S1 Fig). DARTs with A32, 7B2, VRC01, or 10E8 arms bound efficiently to both cell lines, while DARTs with PGT145 or PGT121 arms bound only to the CM244 Env-expressing cell line (moderately for the PGT145 arm and weakly for the PGT121 arm). These latter binding patterns appear to reflect attenuated recognition of one or both of these particular Env isolates by the PGT121 in the DART format, because the parental antibodies bind efficiently to cells expressing Env from other HIV isolates (S1B Fig) [45–47]. In summary, our data demonstrate that all 6 HIVxCD3 DARTs exhibit binding to CD3 and Env (in the form of monomeric gp140 and/or cell surface Env), and the differential binding pattern across different systems is likely related to the ability of specific DARTs to recognize Env antigens from different HIV strains.

### HIVxCD3 DARTs Induce Potent CD8 T Cell-Dependent Killing of Resting CD4 T Cells Infected with Multiple HIV Isolates

**Fig 3:**
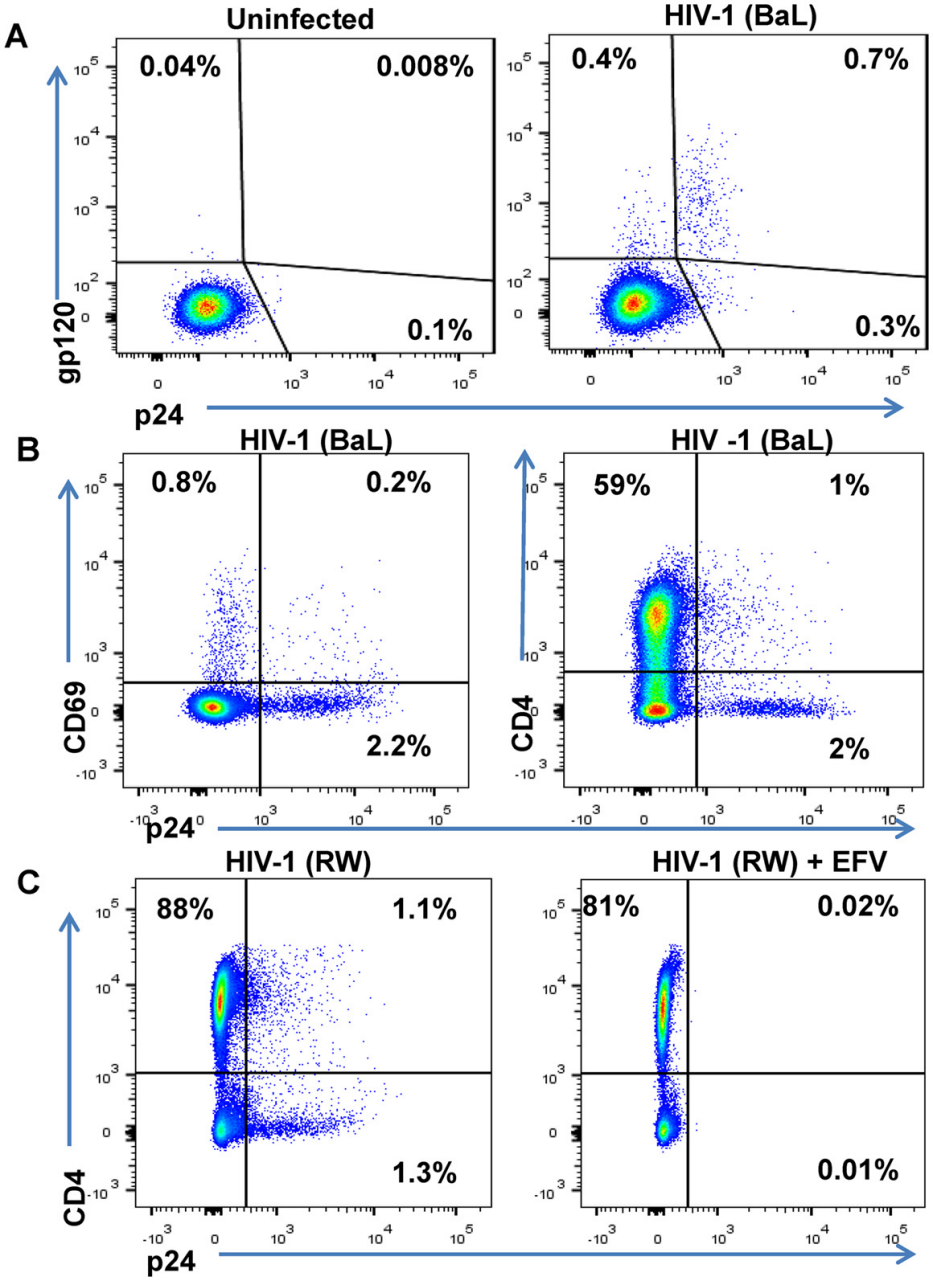
Phenotype of unstimulated primary CD4 T cells infected with HIV in vitro. Unstimulated primary CD4 T cells isolated from a healthy human participants’ PBMCs were spinfected with HIV-1 isolates. (A) FACS results are depicted from representative participant’s cells that were mock-infected or spinfected with HIV-1 (BaL strain) [49]. After 6 days in culture, cells were surface-stained with biotinylated PGT121 Ab and then permeabilized, fixed, and stained with anti-p24 Ab [48]. (B) HIV-1 BaL-infected cells were incubated for 6 days, stained with anti-CD69 Ab and anti-CD4 Ab and then permeabilized, fixed, and stained with anti-p24 Ab. (C) Unstimulated primary CD4 T cells isolated from a healthy participant’s PBMCs were spinfected with HIV-1 RW and incubated in the absence or presence of the antiretroviral efavirenz (EFV) at 1.5 μM. After 5 days of culture, cells were stained with anti-CD4 and anti-p24 Ab and quantified by FACS. The percentages of viable cells for each staining are indicated.

The DARTs were evaluated in a FACS-based cytotoxicity model to assess their ability to mediate CD8 T cell-dependent killing of HIV-infected cells [48]. A resting CD4 T cell in vitro model of HIV infection was developed to create targets that are not actively dividing, as latently infected cells are likely to be in a resting state in vivo [49,50]. Briefly, unstimulated primary CD4 T cells purified from healthy human participants’ peripheral blood mononuclear cells (PBMCs) were spinfected with replication-competent HIV isolates. After 6 to 7 days in culture, typically 1% to 3% of the total CD4 population expressed intracellular p24 and surface Env proteins (Fig 3A). HIV-infected cells exhibited a resting phenotype, indicated by a low level of CD69 surface expression (Fig 3B). Furthermore, HIV-infected cells exhibited decreased expression of CD4, consistent with Nef-mediated CD4 down-regulation [51]. Detection of surface Env expression by FACS required biotinylation of a high-affinity anti-Env mAb (e.g., PGT121), suggesting that the HIV-infected target cells in this model have relatively low levels of Env expression, which we consider a key attribute for relevant testing of the Env-targeting mechanisms. Addition of cART at the time of infection inhibited the expression of HIV proteins at day 6 post-infection, indicating that the FACS detected de novo viral protein expression rather than the presence of virions from the inoculum on the surface of CD4 T cells (Fig 3C).

To generate effector cells, autologous unstimulated CD8 T cells were purified from the same participant’s PBMCs as the CD4 T cells infected with HIV. CD8 T cells were then co-cultured with HIV-infected CD4 T cells at varying ratios and duration in the presence or absence of HIVxCD3 DARTs ranging from 0.6 pM to 2,000 pM. The percentage of DART-induced cytotoxicity was determined by measuring the reduction in CD8- p24+ cells with an active (HIVxCD3) or control (RSVxCD3) DART compared with a no DART control (S2 Fig). HIVxCD3 DART-mediated killing of HIV-infected CD4 T cells plateaued at a CD8:CD4 T cell ratio of 2:1 after a co-culture period of 72 hours (S3 Fig). The kinetics of DART-dependent CD8 T-cell killing of HIV-infected CD4 T cells are similar to those reported for bispecific molecules that redirect resting T-cells at low E:T ratios against other targets [52,53]. HIVxCD3 DARTs-mediated killing of HIV-infected CD4 T cells required the presence of CD8 T cells, and the control RSVxCD3 DART did not induce any appreciable killing of HIV-infected CD4 T cells (S2 Fig). HIVxCD3 DARTs did not mediate killing of uninfected p24-negative CD4 T cells (S2 Fig). To confirm that the reduction in percent p24-positive CD4 T cells mediated by HIVxCD3 DARTs was indicative of death of HIV-infected cells, both cell-associated vRNA and vDNA were measured in cell pellets that were collected at the same time as p24 measurements. Reduction in the percent p24-positive CD4 T cells correlated well with the reduction in the number of vRNA-positive and vDNA-positive cells co-cultured with HIVxCD3 DARTs but not with RSVxCD3 control DART (S4 Fig). The reduction in percent p24-positive CD4 T cells was slightly larger than the reduction in cell-associated vRNA or vDNA, which may indicate that a fraction of HIV RNA and DNA-positive cells express sufficient Env protein to be recognized and killed by HIVxCD3 DARTs. Analysis of cell culture media confirmed that DARTs remained stable under the cell killing assay conditions (S5 Fig).

**Fig 4:**
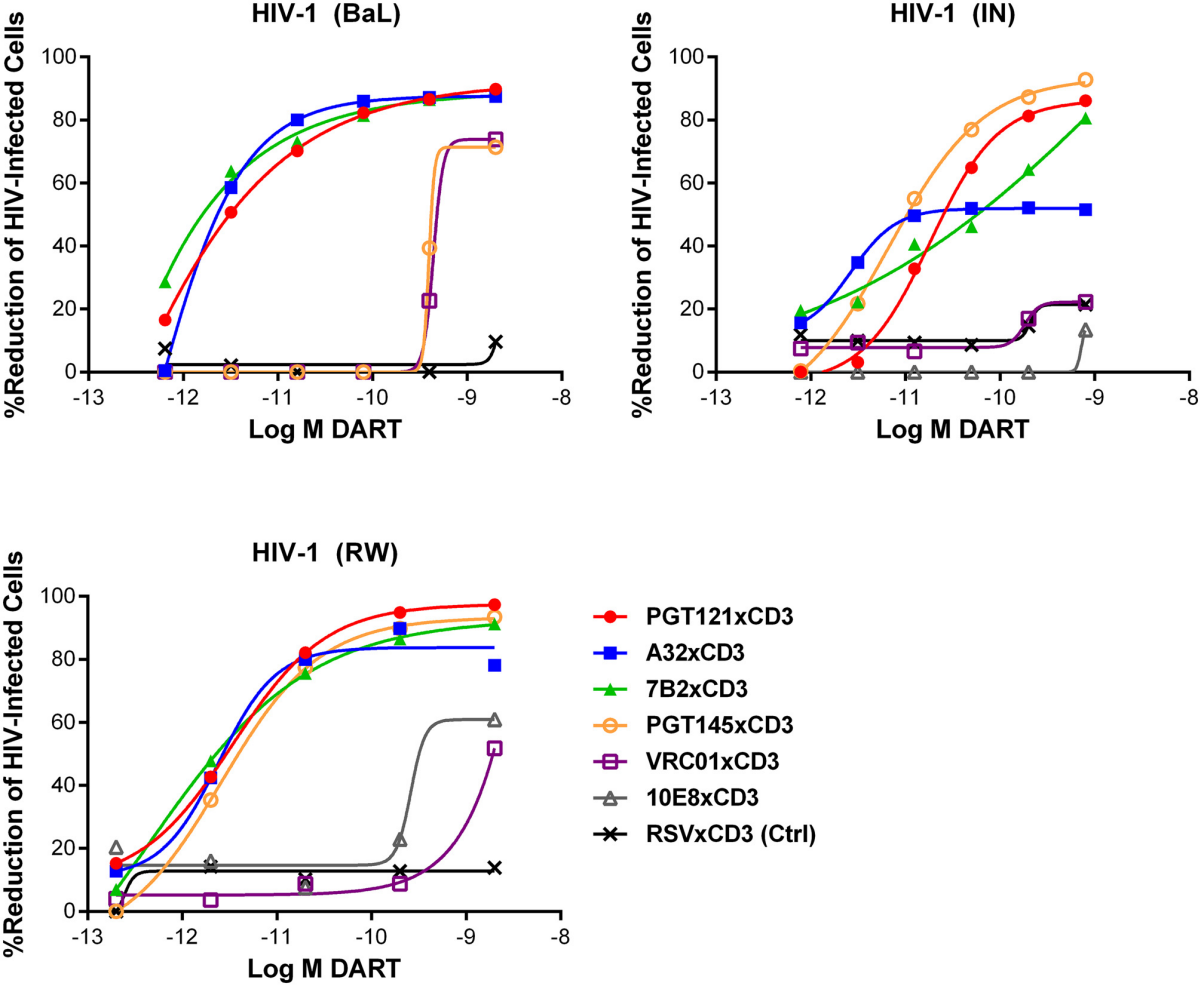
HIV DARTs induce CD8 T cell-dependent cytolysis of CD4 T cells infected with diverse HIV-1 isolates in vitro. Unstimulated CD4 T cells were infected with HIV-1 RW, IN, or BaL isolates and co-cultured with autologous CD8 T cells in the absence or presence of active DARTs (HIVxCD3) or control DART (RSVxCD3) at concentrations ranging from 0.6 to 2,000 pM and at a CD8 T cell:CD4 T cell ratio of 2:1 for 72 hours. Cytolytic activities were determined as described in Materials and Methods. HIVxCD3 DARTs with 6 different Env-specificities were evaluated. There was no appreciable killing mediated by 10E8xCD3 or VRC01xCD3 DARTs. Representative data with cells from a single participant are depicted. Results from multiple participants are summarized in Table 1.

HIVxCD3 DART constructs derived from PGT121, 7B2, and A32 demonstrated potent killing of CD4 T cells infected with each of the three tested diverse CCR5-tropic HIV-1 isolates with divergent envelope sequences: BaL, IN/93/905 (IN), and 92/RW/008 (RW) (Fig 4). A32xCD3 showed the most consistent potency for inducing redirected killing activity with EC_50_ values of 4.2 to 5.4 pM across all three HIV isolates (Table 1). On the other hand, PGT121xCD3 induced more consistent maximal killing activity with E_max_ values of 86–93% (Table 1). PGT145xCD3 potently killed IN and RW, but was less potent against BaL. These findings are consistent with the ability of the parental PGT145 IgG to potently neutralize IN and RW but not BaL (S6 Fig). In contrast to these HIVxCD3 DARTs, 10E8xCD3 and VRC01xCD3 did not potently kill CD4 T cells infected with the HIV isolates that were tested. The parental 10E8 IgG and VRC01 IgG did bind HIV BaL-infected cells (S1B Fig). In addition, VRC01 IgG neutralized each HIV isolate tested, while 10E8 IgG weakly neutralized two of the three isolates (S6 Fig). In summary, HIVxCD3 DARTs containing CDRs from PGT121, PGT145, A32, or 7B2 mediated picomolar killing at levels > 90% in a model with primary unstimulated CD4 T cells infected with multiple HIV isolates.

**Table 1:** Summary of HIVxCD3 DART-mediated killing of HIV-1 infected CD4 T cells in vitro.

HIV-1	A32xCD3			7B2xCD3			PDG121xCD3			PGT145xCD3		
	EC_50_ (SD)	%Max (SD)	n	EC_50_ (SD)	%Max (SD)	n	EC_50_ (SD)	%Max (SD)	n	EC_50_ (SD)	%Max (SD)	n
BaL	5.4 (4.4)	86.1 (3.5)	5	1.9 (13.4)	82.5 (10.5)	4	0.2 (4.7)	90.3 (4.5)	6	446.7 (81.7)	70.1 (14.3)	3
IN	4.2 (5.4)	53.6 (6.8)	4	20.5 (215.6)	74.6 (14.6)	3	24.0 (36.9)	86.1 (8.2)	6	7.3 (17.6)	91.2 (2.7)	4
RW	4.4 (3.1)	87.8 (7.0)	3	4.4 (3.7)	87.0 (5.9)	2	4.4 (3.2)	93.3 (6.2)	3	1.8 (1.5)	85.6 (11.8)	3

n = number of participants;

SD = standard deviation.

Values are geometric means of EC_50_ (pM) and maximal killing (%Max).

10E8 and VRC01 DARTs had low potency, with EC_50_ values >1 nM.

**Fig 5:**
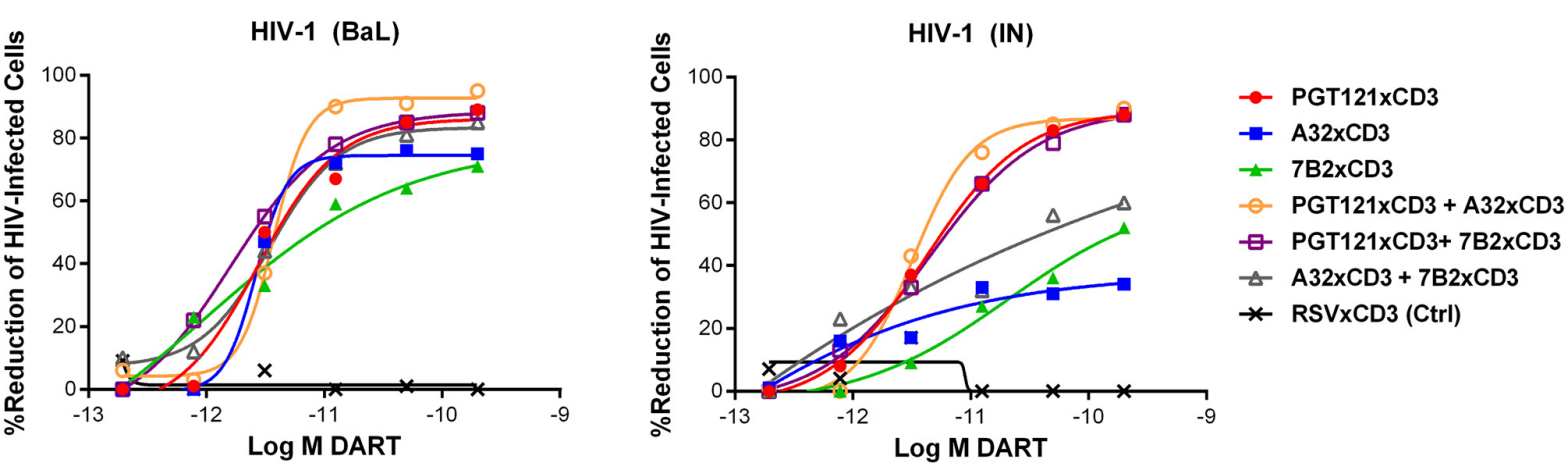
Combinations of HIVxCD3 DARTs induce CD8 T cell-dependent cytolysis of CD4 T cells infected with HIV-1 in vitro. Unstimulated CD4 T cells were infected with HIV-1 BaL or IN and co-cultured with autologous CD8 T cells at a CD8 T cell:CD4 T cell ratio of 2:1 for 72 hours in the presence of indicated individual DARTs or DART combinations. Cytolytic activities were determined as described in Materials and Methods. Representative data with cells from a single participant are depicted. Results from multiple participants are summarized in Table 2.

Combinations of HIVxCD3 DARTs were profiled with the goal of maximizing recognition of diverse Env antigens expected to be present in participants infected with various HIV strains and subtypes. Pairwise combinations of PGT121xCD3, A32xCD3, and 7B2xCD3 were evaluated against BaL- and IN-infected cells. These three DART constructs performed well individually and would not be expected to compete for binding to Env, as they recognize spatially distinct Env epitopes (Fig 1B). For PGT121xCD3 paired with either A32xCD3 or 7B2xCD3, the combined potency and maximal level of killing observed for both HIV isolates were not substantially different from the effect of PGT121xCD3 alone, while the A32xCD3 plus 7B2xCD3 combination was slightly less effective against the CD4 T cells infected by the HIV IN isolate (Fig 5). For any of the tested pairs, there was no apparent synergistic benefit of the combination, a result that may be due to the very high potency and level of killing already achieved with each HIVxCD3 DART alone.

**Table 2:** Summary of HIVxCD3 DART combination-mediated killing of HIV-1 infected CD4 T cells in vitro.

HIV-1	PGT121xCD3		7B2xCD3		A32xCD3		PDG121xCD3 + 7B2xCD3		PGT121xCD3 + A32xCD3		7B2xCD3 + A32xCD3	
	EC_50_ (SD)	%Max (SD)	EC_50_ (SD)	%Max (SD)	EC_50_ (SD)	%Max (SD)	EC_50_ (SD)	%Max (SD)	EC_50_ (SD)	%Max (SD)	EC_50_ (SD)	%Max (SD)
BaL	3.8 (1.7)	89.4 (4.9)	2.5 (3.7)	82.7 (36.1)	2.5 (0.1)	60.0 (19.1)	2.9 (2.6)	87.9 (14.8)	3.9 (0.3)	88.0 (1.4)	3.8 (0.5)	70.3 (7.8)
IN	7.3 (6.2)	89.4 (6.4)	50.2 (70.4)	76.9 (13.4)	1.2 (31.5)	84.6 (31.8)	8.7 (8.7)	92.0 (2.8)	7.3 (9.5)	88.5 (2.1)	19.1 (18.6)	55.3 (6.4)

SD = standard deviation.

Values, n = 3, are geometric means of EC_50_ (pM) and maximal killing (%Max).

### HIVxCD3 DARTs Can Reduce Viral Production Ex Vivo from HIV-Infected Participants on Suppressive cART

The in vitro model of HIV infection used above reproduces an important aspect of HIV reservoirs in that it uses resting CD4 T cells, but it cannot fully recapitulate the latency that is found in participants treated with suppressive cART. Importantly, HIV-infected cells derived from participants on cART are likely to be less frequent and express lower levels of HIV proteins, if any, compared with the resting cells infected in vitro. These biological differences make it critical to evaluate the efficacy of HIVxCD3 DARTs ex vivo using cells isolated from HIV-infected participants with prolonged virus suppression by cART. We used two different models to determine whether DARTs can impact HIV-infected cells isolated from participants. In the first model, we determined whether HIVxCD3 DARTs could reduce HIV virion production from unstimulated cells from HIV-infected cART-suppressed participants. This resting ex vivo model was designed to represent how HIVxCD3 DARTs may be initially evaluated alone in vivo, similar to the Phase 1 trial with bNAb 3BNC117 [54]. In the second model, we tested whether treatment with HIVxCD3 DARTs in combination with a latency reversal agent can affect the response of the viral reservoir to re-stimulation with the same agent. This stimulated model was designed to represent how HIVxCD3 DARTs could be evaluated in combination with a compound that enhances HIV protein expression.

**Fig 6:**
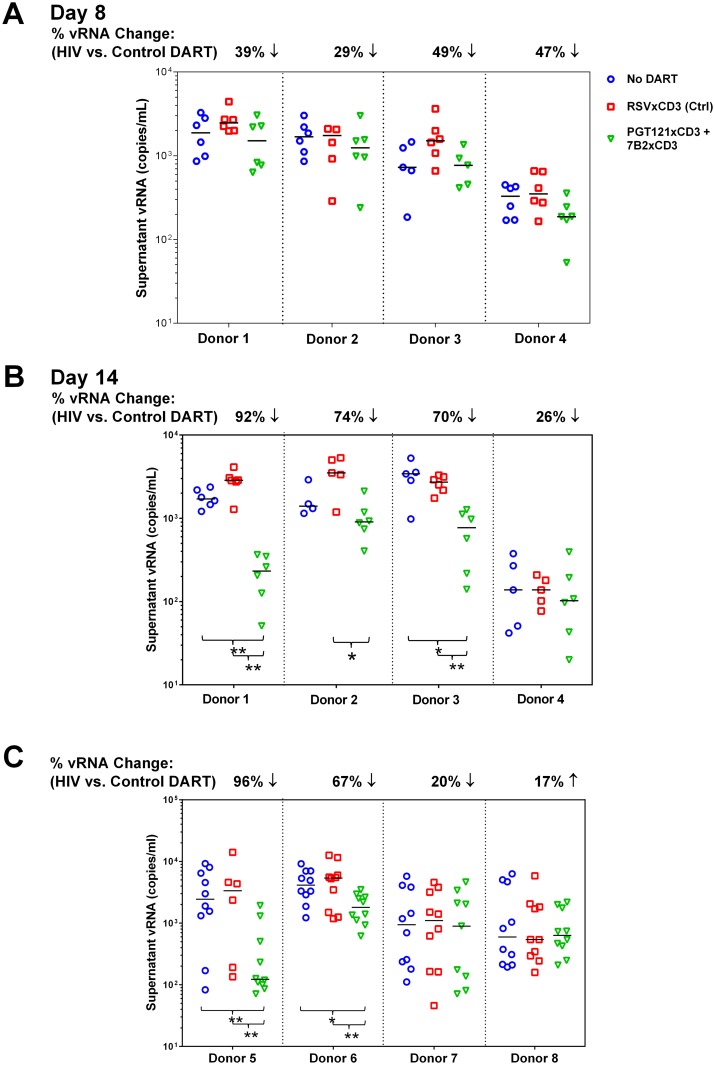
HIVxCD3 DARTs reduce ex vivo HIV expression in PBMCs isolated from HIV-infected participants on suppressive cART. (A-B) Resting Model: Unstimulated PBMCs from 4 HIV-infected participants on suppressive cART were cultured with 400 pM of a DART combination (200 pM each of PGT121xCD3 and 7B2xCD3) or 400 pM of RSVxCD3. Supernatant HIV RNA was quantitated on Day 8 (A) and Day 14 (B). By Day 14, statistically significant reduction in vRNA level was observed in 3 out of 4 participants treated with the HIVxCD3 DART combination, but not with control DART compared to no DART (*p<0.05, **p<0.005, 2-tailed Mann-Whitney U-Test). Horizontal bars represent the medians for each group. (C) PKC Agonist-Inducible Model. PBMCs from 4 HIV-infected participants on suppressive cART were cultured untreated or treated with 1 μM indolactam, a PKC agonist, and with or without an HIVxCD3 DART combination (200 pM PGT121xCD3 + 200 pM 7B2xCD3) or control RSVxCD3 DART (400 pM). After 7 days of incubation, total CD4 T cells were isolated from PBMCs and re-stimulated with 1 μM indolactam. After an additional 3 days of incubation, supernatant HIV RNA was quantitated. In 2 out of 4 participants, HIVxCD3 DARTs significantly reduced the indolactam-induced vRNA vs. control DART or no DART (**p<0.005, *p<0.05, 2-tailed Mann-Whitney U-Test). Horizontal bars represent the medians for each group.

PBMCs were isolated from cART-treated HIV-infected participants whose viral load was undetectable for a minimum of 12 months. PBMCs were used to evaluate DARTs ex vivo rather than purified CD4 and CD8 T cells. PBMCs may more closely mimic the biological diversity of the reservoir and ratios of effector T cells, and PBMCs do not require additional purification steps that reduce the yield of limited materials. In the unstimulated ex vivo model, a combination of PGT121xCD3 and 7B2xCD3 DARTs or the RSVxCD3 control DART was added to PBMCs in the absence of any activating agent. Cultures were maintained in the presence of antiretroviral agents (ARVs) to prevent HIV transmission to uninfected cells, and levels of supernatant vRNA were quantified by qRT-PCR after 8 days and 14 days of culture (S7 Fig). Supernatant vRNA was measured as a functional indication of virion production to assess the impact of DARTs on the HIV reservoir. Using this model, we have previously demonstrated that supernatant HIV RNA can be pelleted by ultracentrifugation, indicating that this HIV RNA is primarily contained within virions [55]. On Day 8 of culture, HIVxCD3 DARTs reduced vRNA supernatant levels by 29% to 49% compared with RSVxCD3 control DART in 4 of 4 participants. These reductions did not reach statistical significance (Fig 6A). By Day 14, HIVxCD3 DARTs reduced supernatant vRNA levels by 26% to 92% in 4 of 4 participants. In 3 participants, these reductions did reach statistical significance (Fig 6B). These ex vivo data suggest that a portion of the HIV reservoir may express sufficient basal levels of Env to enable targeting by HIVxCD3 DARTs.

In the stimulated ex vivo model, a combination of two HIVxCD3 DARTs or the control RSVxCD3 DART were added to PBMCs that were untreated or activated with the PKC agonist indolactam, which robustly activated HIV in all of the participants tested (S8A Fig). After 6 days, CD4 T cells were isolated from PBMCs and activated with indolactam for an additional 3 days (S7 Fig). This re-stimulation model was designed to measure the effect that HIVxCD3 DARTs may have had on the inducible HIV reservoir. In 2 of 4 participants that were tested, the HIVxCD3 DARTs significantly reduced the level of supernatant HIV RNA that was induced following the re-stimulation with indolactam, while the control DART had no appreciable effect on the re-stimulation of inducible reservoir (Fig 6C). In 4 additional participants that were tested in this model, the initial stimulation with indolactam was sufficient, in the absence of DARTs, to reduce the level of supernatant HIV RNA induced by subsequent re-stimulation with indolactam. The addition of HIVxCD3 DARTs or control DART did not demonstrate additional benefit to reduce the inducible reservoir response in these participants (S8B Fig). Taken together, the results from these ex vivo studies indicate that HIVxCD3 DARTs are capable of targeting either unstimulated or stimulated primary HIV-infected cells from a subset of virologically suppressed participants.

### Extended Half-Life HIVxCD3 DARTs Redirect CD8 T Cells to Kill HIV-Infected CD4 T Cells

**Fig 7:**
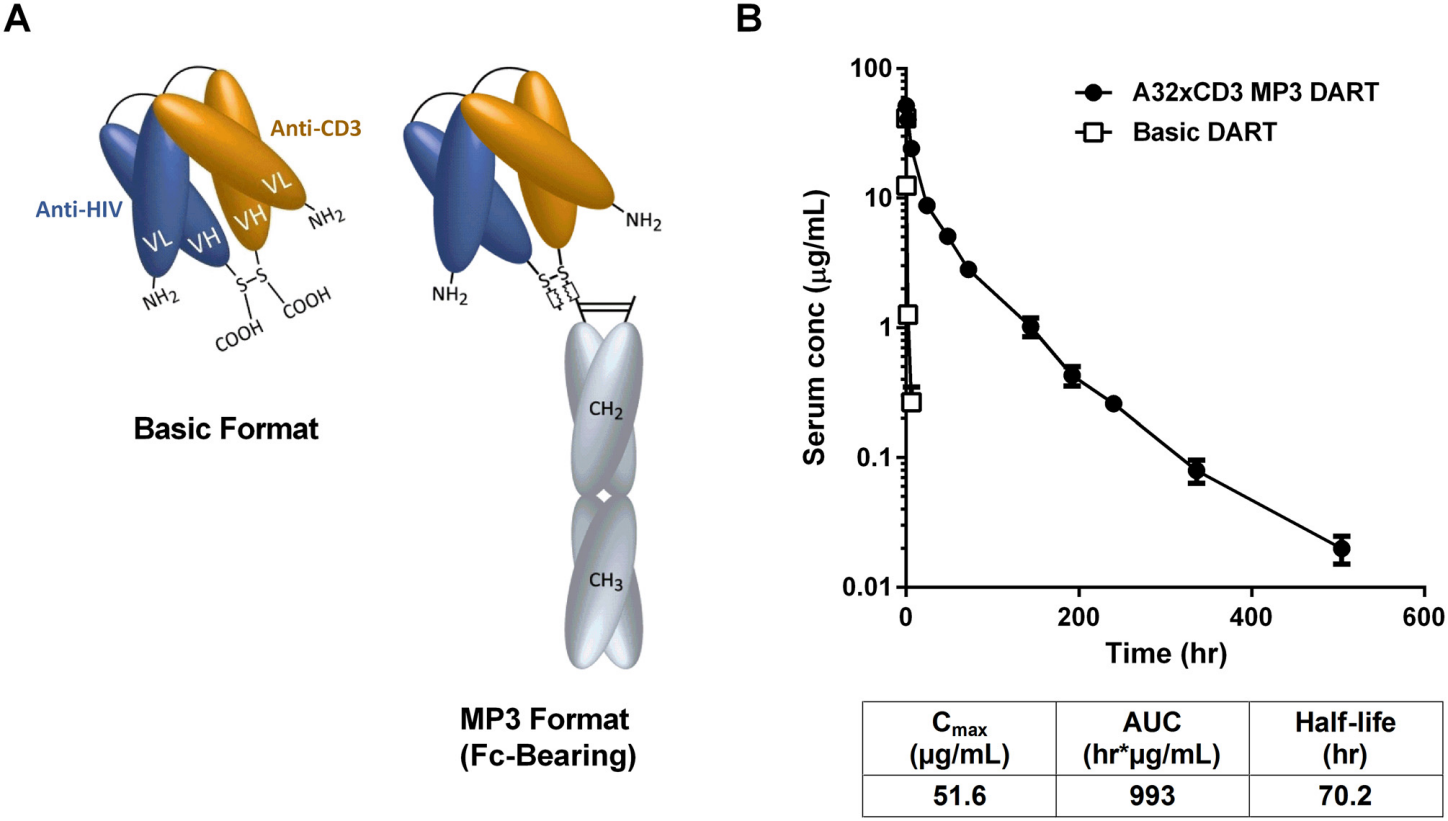
HIVxCD3 DARTs in basic and MP3 format. (A) Schematics of DARTs in basic and MP3 (Fc-bearing) format are shown. The anti-HIV and anti-CD3 domains are colored in blue and orange, respectively, and the human IgG1 Fc domains are shaded in grey. The chains of the Fc domain are modified to contain L234A/L235A mutations that inhibit Fc binding to activating FcγRs. The chains of the Fc domain do not inhibit binding to FcRn, which prolongs serum half-life and exposure. (B) Pharmacokinetic profile in human FcRn transgenic mice. The A32xCD3 MP3 DART was administered at 5 mg/kg by iv injection and serum concentrations were measured over time (closed circles). For comparison, the serum concentration-time curve for a basic DART with different specificities is shown (open squares). The PK parameters for the A32xCD3 MP3 DART are presented in the table.

The MP3 DART format was developed to prolong the short circulating half-life of basic format DARTs [28]. MP3 DARTs contain a human IgG1 Fc domain that has been mutated (L234A/L235A) to inactivate effector function via binding to FcγRs and/or complement, while retaining binding to the neonatal FcR (FcRn) to engage the IgG salvage pathway (Fig 7A) [56,57]. The MP3 DART format would likely be preferable for clinical applications, as it would reduce dosing frequency while maintaining optimal exposure levels.

A total of four HIVxCD3 DARTs containing Env binding CDRs derived from A32, 7B2, PGT121, and PGT145 were constructed in MP3 format, produced in CHO cells and purified. The formation of properly assembled molecules was confirmed by reducing and non-reducing SDS-PAGE and analytical SEC; the average purity of the assembled HIVxCD3 MP3 DART molecules was 96%. The binding properties of these four HIVxCD3 MP3 DARTs and the matching control RSVxCD3 MP3 DART were evaluated. Binding to recombinant CD3 and JRFL gp140 were slightly reduced for the MP3 DARTs compared with the corresponding basic DARTs (S9A, S9B and S9C Fig). Similarly, binding to the surface of HEK293 cells expressing HIV CM244 env was slightly reduced with the MP3 DARTs compared with the same corresponding basic DARTs (S9D and S9E Fig). However, when administered to human FcRn transgenic mice, a model that offers reliable predictions of antibody pharmacokinetics in monkeys and humans [58,59], the A32xCD3 MP3 DART exhibited a major improvement in serum half-life and exposure compared with a DART in basic format, which was cleared in only a few hours (Fig 7B). The pharmacokinetic parameters for the A32xCD3 MP3 DART in human FcRn transgenic mice approximate those observed with IgG1 molecules.

**Fig 8:**
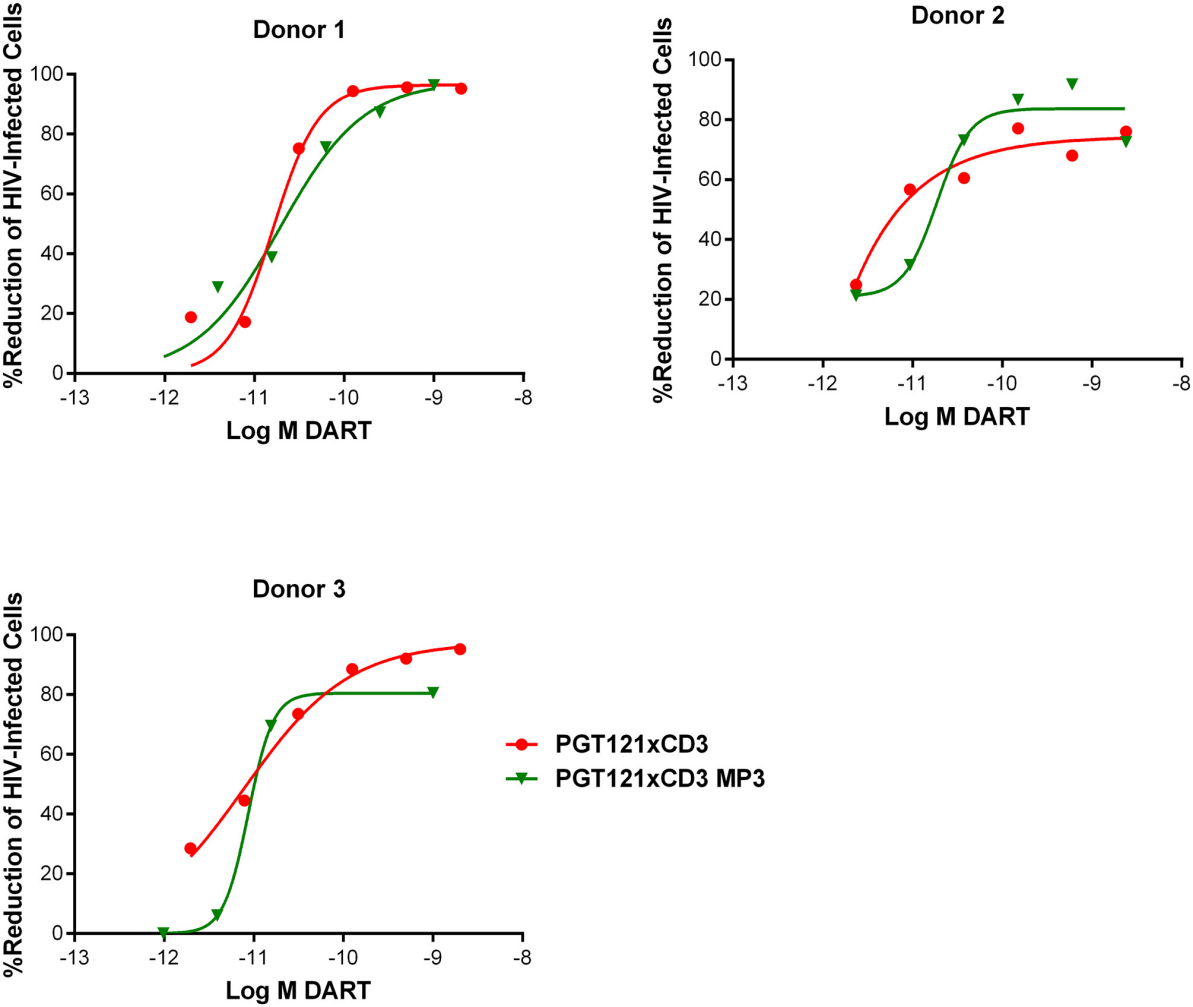
HIVxCD3 DARTs in MP3 format and basic format induce the CD8 T cell-dependent killing of HIV-Infected CD4 T cells in vitro with comparable potency. Unstimulated CD4 T cells were infected with HIV-1 BaL and co-cultured with autologous CD8 T cells at a CD8 T cell:CD4 T cell ratio of 2:1 for 72 hours in the presence of either a regular format PGT121xCD3 DART or an extended half-life MP3 format PGT121xCD3 DART. After 72 hours of co-culture, the % reduction in p24-positive CD4 T cells for each condition relative to no DART control were determined by FACS. Representative data from a single participant are depicted.

HIVxCD3 DARTs in MP3 and basic format were compared for their ability to mediate redirected CD8 T cell-dependent killing of HIV-infected cells. PGT121xCD3 DARTs in basic and MP3 format were compared side-by-side in the in vitro model of resting CD4 T cell infection using cells prepared from three independent participants. Importantly, both DART formats exhibited similar potency and maximum elimination of HIV-infected cells (Fig 8). These results support further investigation of the extended half-life MP3 DART format.

### HIVxCD3 DARTs Do Not Increase HIV Spread during Productive Infection

A bi-specific Ab that simultaneously targets HIV Env and CD3 expressed on CD4 T cells has the potential to enhance cell-to-cell spread of HIV by binding Env on an HIV-infected cell and CD3 on an adjacent uninfected CD4 T cell. This interaction could activate uninfected cells and, in turn, make them more susceptible to cell-to-cell viral transmission. To evaluate the potential for HIVxCD3 DARTs to activate CD4 T cells, we measured the expression of the cell surface activation markers CD25, CD69, and HLA-DR on the uninfected p24-negative and HIV-infected (p24-positive) CD4 T cells following HIV infection in the presence of DARTs. In uninfected and HIV-infected CD4 T cells, PGT121xCD3 MP3 DART increased the frequency of CD69-positive and CD25-positive CD4 T cells, but not HLA-DR-positive CD4 T cells compared with the RSVxCD3 MP3 control DART (S10A Fig). To evaluate the potential for this partial activation to contribute to cell-to-cell HIV spread, we developed two in vitro infection models using primary CD4 T cells that were either unstimulated or activated. In an activated CD4 T cell spreading infection model, the PGT121xCD3 MP3 or A32xCD3 MP3 DART increased neither the frequency of HIV-infected cells nor virus production following incubation for 5 days. On the contrary, PGT121xCD3 MP3, but not A32xCD3 MP3, profoundly reduced the fraction of p24-positive cells in the activated model of cell-to-cell transmission, a finding that is consistent with the neutralizing ability of PGT121 (S10B Fig). These data indicate that HIVxCD3 DARTs derived from bNAbs can exert a direct antiviral effect in the absence of effector CD8 cells likely by neutralizing virus or blocking cell-to-cell spread. In the resting model of HIV infection, there was no evidence for enhanced viral replication in the presence of PGT121xCD3 MP3 when compared with the RSVxCD3 MP3 control DART (S10C Fig). Taken together, these results indicate that HIVxCD3 DARTs are unlikely to pose a risk for enhancing the spreading of HIV infection, and the constructs derived from neutralizing antibodies may, in fact, protect uninfected cells.

## Discussion

Despite the established clinical efficacy of suppressive cART, HIV reservoirs persist throughout the life of treated participants. In longitudinal studies, HIV sequence stability suggests that cART effectively prevents active virus replication and spread [60]. cART does not eliminate HIV-infected cells or prevent HIV antigen expression, and persistent HIV antigen exposure may contribute to chronic immune dysfunction and accelerated non-AIDS diseases [61]. Therefore, there is a clear unmet medical need to eliminate HIV reservoirs. Reduction of reservoirs to levels that restore normal immune function may also provide a health benefit. Several HIV cure strategies target HIV Env because it is selective and may be exposed on the surface of infected cells. Env-targeting bNAbs have been able to reduce viral reservoirs in preclinical models of persistent HIV infection, as measured by declines in proviral DNA and delays in viral rebound [23,24,62,63]. bNAbs have the potential to eliminate HIV-infected cells via FcγR-mediated effector functions that engage immune effector cells, such as monocytes, macrophages, natural killer cells, and neutrophils [64–67]. The success of bNAbs in animal studies has stimulated interest in conducting investigational clinical trials aimed at targeting Env in HIV-infected participants using either passively administered IgG, e.g., VRC01, sustained in situ production of bNAbs by AAV-vectored approaches, e.g. PG9, or vaccination approaches designed to elicit bNAbs [68].

CD3-targeted bi-specific DARTs are antibody-based molecules that elicit a cell-mediated killing mechanism that is distinct from Fc effector-competent bNAbs. HIVxCD3 DART molecules specifically redirect cytotoxic T cells to Env-expressing target cells and induce their lysis. This process involves simultaneous binding to the surfaces of HIV-infected cells and CD3-expressing polyclonal T cells by the anti-Env and anti-CD3 arms, respectively. Bi-specific antibody-mediated redirected T cell killing of target cells is concomitant with effector T cell activation, proliferation, and upregulation of granzyme B and perforin in a target-dependent manner [28,69], which may prime CTL for serial cytotoxicity [53]. These bi-specific T-cell redirecting molecules are effective in vivo at doses many-fold lower than those typically employed for mAbs [70]. For example, blinatumomab, a CD19xCD3 BiTE, has been shown to be clinically potent and efficacious with an acceptable safety profile when administered at doses of 28 μg/day and was approved in 2014 for the treatment of relapsed or refractory B-precursor acute lymphoblastic leukemia [26]. In nonhuman primate studies with DARTs, treatment with a CD123xCD3 basic DART at doses ranging from 0.1 to 1.0 μg/kg/day resulted in profound depletion of circulating CD14^-^ CD123^+^ cells, the intended target cells [28], and treatment with a CD19xCD3 MP3 DART at doses of 5–10 μg/kg/week resulted in profound depletion of CD19^+^ cells in the periphery and in lymphoid tissues [57]. While anti-drug antibody (ADA) responses against DARTs have been observed in these cynomolgus monkey studies, the frequencies appear to be comparable to those observed with other human or humanized monoclonal antibodies in monkeys, and, importantly, it is well established that immunogenicity in cynomolgus monkeys is not predictive of immunogenicity in human subjects [71]. The immunogenicity of DARTs for oncology and autoimmune indications in human subjects is being monitored in multiple clinical studies (NCT02152956, NCT02248805, NCT02376036, NCT02454270).

DARTs have inter-chain disulfide bonds at their C-termini and are structurally compact and well suited for forming stable cell-to-cell contacts between CTL and target cells. Additionally, DARTs exhibit greater potency than BiTEs in side-by-side comparisons [30,72]. The enhanced potency of bi-specific DARTs may be particularly relevant in regards to HIV reservoirs, which represent a low-frequency target that likely express Env at low densities [73]. Furthermore, the importance of CD8 CTL in controlling the HIV reservoir is evident in elite controllers, who have demonstrated the ability to suppress viremia in the absence of cART [48,74,75]. Unlike therapeutic HIV vaccine strategies that can only enhance HIV-specific CTL, bi-specific HIVxCD3 DARTs can redirect polyclonal CTL to kill HIV-infected cells. This is an important distinction because HIV-specific CTL in some cART-treated participants may have an anergic or senescent phenotype characterized by defects in cytotoxic function [76,77]. This would also be useful in cases where viral epitopes have evolved to escape HIV-specific CTL killing, as is typically the case for cART-suppressed HIV participants initially treated during chronic infection [78].

Here, we report that HIVxCD3 DARTs with different Env specificities elicit potent and specific elimination of HIV-infected cells. For these studies, we utilized an in vitro model based on the infection of unstimulated primary CD4 T cells with wild-type HIV isolates [12,50]. As opposed to dividing cell lines or mitogen-stimulated latency models, a resting primary cell model may better approximate the resting state and corresponding low levels of surface Env expressed on reservoir cells from HIV-infected participants on cART. Primary unstimulated CD4 T cells may also respond to killing signals from effector cells in a more relevant manner than activated CD4 T cells. For these same reasons, the effector cells used in this model were unstimulated, autologous CD8 T cells. Multiple Env-specificities were evaluated in our in vitro model to determine whether the spatial location of the Env epitope might influence the efficiency of redirected lysis. Bi-specific constructs were derived from both bNAbs and broadly reactive, non-neutralizing Abs. These classes likely recognize different Env forms, and it is unclear whether neutralization activity is preferred for recognizing cellular membrane forms of Env and inducing efficient redirected lysis [43,79]. bNAbs selected for these studies recognize spatially distinct Env domains [80] and are some of the broadest, most potent mAbs available (PGT121, V3-glycan; PGT145, V1/V2 loop [32]; VRC01, CD4bs [81]; 10E8, gp41 [82]). DARTs with bNAbs-derived arms may preferentially, or in some cases exclusively, e.g., PGT145, bind the mature Env trimers [31,83].

In our in vitro HIV infection model, PGT121- and PGT145-derived HIVxCD3 DARTs exhibited higher maximal killing and more potent killing of infected cells than those derived from VRC01 and 10E8 (Fig 4 and Table 1). VRC01xCD3 and 10E8xCD3 DARTs were able to bind Env-transfected cells (S1A Fig), and in single cycle neutralization assays, the parental VRC01 IgG, but not 10E8 IgG, demonstrated potent neutralization of the HIV isolates tested in the cytotoxicity assays (S5 Fig). Neutralization of an HIV isolate by the parental IgG may not correlate with corresponding DART cytolytic activity, given that virions and infected cells may express different forms and levels of Env. Recognition and binding of Env is required, but it may not be sufficient for potent DART-mediated killing. For example, VRC01 and 10E8 IgGs each bound Env on HIV-infected cells (S1B Fig), suggesting that spatial conformation of certain Env epitopes, and the corresponding geometry of the DART-mediated synapses that form, may determine cytolytic activity in some cases. In our model, DARTs targeting the V1V2 domain or the V3 glycan domain of Env induced more efficient redirected lysis than DARTs targeting the CD4 binding site or MPER domains. However, it is premature to generalize these results before testing additional antibodies from each Env domain class against a larger number of HIV isolates.

In contrast to bNAbs, HIVxCD3 DARTs with arms derived from broadly reactive, non-neutralizing mAbs, such as A32 and 7B2, may be expected to preferentially recognize their epitopes in the context of nonfunctional forms of Env [43,46,79,84]. For example, 7B2 preferentially binds gp41 when gp120 dissociates [82], and A32 binds to a CD4-induced epitope that is exposed on the functional Env trimer only after CD4 binding during entry [40]. However, the A32 epitope (C1 domain) is expressed on the surface of infected cells early and thus may be an efficient ADCC epitope [37]. In the RV144 vaccine trial, for example, A32 blocked ADCC activity in 96% of cases where ADCC was induced, suggesting that potentially protective ADCC responses were directed to epitopes preferentially exposed on non-functional Env forms [85,86]. Nonfunctional Env forms may in fact be the predominant Env expressed on infected cells, as fully cleaved, trimeric forms accounted for only 10% of total cellular Env for 3 HIV isolates examined [82]. This may partially explain why DARTs derived from the non-neutralizing Abs 7B2 and A32, which that target nonfunctional Env forms, bound to HEK293-D371 and HEK293-D375 with higher MFIs than DARTs derived from the bNAbs PGT121 and PGT145 (S1 Fig). Notably, A32xCD3 and 7B2xCD3 DARTs both exhibited potent and robust killing of HIV-infected primary resting CD4 T cells that was comparable to those of PGT121xCD3 and PGT145xCD3 DARTs (Fig 4 and Table 1).

Given the ability of HIVxCD3 DARTs to kill HIV-infected cells in vitro, we evaluated DARTs ex vivo using PBMCs isolated from HIV-infected cART-treated participants with suppressed viral load. PBMCs may better represent the physiological diversity of HIV reservoir and the ratio of target and effector cells. Additionally, material from HIV participants is always limited, and use of PBMCs reduces loss of cells during isolation of individual cell subsets, enabling more robust execution of key experiments by including a larger number of replicates. Ex vivo models have potential advantages compared to in vitro models. For example, HIV-infected cells ex vivo may express more biologically relevant levels and forms of Env. These cells may also respond differently to cytotoxic signaling than cells infected in vitro. In addition, effector CD8 T cells from HIV-infected participants on cART may have altered functions relative to CD8 T cells from healthy individuals. Reduction of cell-associated proviral vDNA is likely a more definitive indication of HIV-infected cell death than reduction in supernatant vRNA. However, available data suggest that it may not be biologically feasible to reduce vDNA in most subjects. For example, sequence data suggest that 88.3% of HIV proviruses in the reservoir of cART-suppressed participants are defective, and maximal activation of resting CD4 T cells in vitro induced infectious virus from <1% of proviruses [87]. In a subsequent study, only 1.5% of proviruses were induced by mitogen to produce virion, as measured by supernatant vRNA [88]. There are also technical difficulties that limit the ability to demonstrate small reductions of vDNA in rare cell populations by quantitative nucleic assays. Prior to ex vivo studies, pair-wise HIV DART combinations were evaluated in vitro to ensure that combinations predicted to increase coverage of diverse strains would not be antagonistic (Fig 5). Given the distinct and complimentary binding properties of bNAbs and broadly reactive, non-neutralizing Abs discussed above, the PGT121xCD3 + 7B2xCD3 combination was selected for ex vivo testing in two models using unstimulated CD4 T cells and CD4 T cells treated with a potent latency reversal agent. Prior to ex vivo evaluation, we demonstrated in vitro that this HIVxCD3 DART combination was capable of reducing cell-associated p24, vRNA, and vDNA (S4 Fig). In the unstimulated ex vivo model, this HIVxCD3 DART combination effectively reduced the level of supernatant vRNA in 3 of 4 participants tested compared with the control DART (Fig 6B). These findings suggest that basal levels of Env expression on unstimulated CD4 T cells may be sufficient for HIVxCD3 DART-mediated reservoir reduction. This result is consistent with the reduction in cell-associated vDNA that was observed in SHIV-infected non-human primates (NHP) treated with ARVs in combination with parental PGT121 [23]. For the stimulated ex vivo model, we selected the PKC agonist indolactam, as it effectively activated HIV in all of the participants tested (S8 Fig). While mitogens, e.g. anti-CD3/anti-CD28 or PMA/ionomycin, may be more effective LRAs than PKC agonists, mitogens can also induce T cell proliferation leading to potential reservoir expansion and/or resistance to DART-mediated cell killing. Our ex vivo stimulated model was designed to mimic conditions similar to those potentially employed in vivo to evaluate HIVxCD3 DARTs. LRAs with mitogenic activity cannot be used in vivo for safety reasons and it is unlikely that an LRA would be available for clinical testing with a level of HIV activation matching that of mitogenic activators. Importantly, PKC agonists as a class have demonstrated the ability to strongly and consistently activate HIV without inducing T cell proliferation [89–92]. In our stimulated ex vivo model, we observed that indolactam treatment combined with HIVxCD3 DARTs reduced the subsequent reactivation of reservoir compared with the control DART in 2 of 4 participants who did not demonstrate a reduced re-stimulation with indolactam alone (Fig 6C). This stimulated model may be less sensitive to reductions if latent viruses that were not activated by the first stimulus are stochastically activated by the second stimulus, as previous work suggests [87]. Additionally, a PKC agonist may not be the optimal latency reversal agent to combine with HIVxCD3 DARTs for reservoir reduction. For example, PKC activation alone may enhance T cell survival by inhibiting apoptosis [93,94]. Additional studies are therefore needed to select optimal latency reversal agents to combine with HIVxCD3 DARTs to enhance elimination of HIV-infected cells. Taken together, results from our ex vivo models demonstrate reduction of virus production that is suggestive of infected cell killing, but ultimate proof of the reservoir reduction would have to be obtained by in vivo testing of DARTs.

In summary, we have demonstrated potent and Env-specific HIVxCD3 DART-mediated killing of HIV-infected cells in vitro and reduction of viral protein expression ex vivo. HIVxCD3 DARTs that target either broadly neutralizing Env epitopes or broadly reactive, non-neutralizing Env epitopes were effective, as was an extended half-life MP3 DART format. Taken together, these results provide support to evaluate this platform in an animal model of HIV latency to determine whether the HIV reservoir can be safely reduced in vivo, as was recently demonstrated by PGT121 IgG in SHIV-infected NHPs.

## Materials and Methods

### Ethics Statement

HIV-infected participants were enrolled into the study at the Quest Clinical Research (QCR) in San Francisco, CA. The study was approved by the Western Institutional Review Board. Informed, written consent was obtained from participants prior to any study procedures.

### Participant Samples

HIV-infected participants participating in the study were selected based on sustained plasma viral load suppression (<50 copies/mL for >12 months), CD4 counts (>350 cells/mL), and absence of co-infection with hepatitis B or C virus.

### Cells and Viruses

HEK293-D371 and HEK293-D375 cell lines with doxycycline-inducible expression of HIV CM244 (subtype AE) gp140 and 92Th023 (subtype AE) gp140, respectively, were obtained from Dr. John Kappes (University of Alabama at Birmingham); cells were maintained in complete RPMI 1640, 20% fetal bovine serum (FBS), 1% Pen/Strep, and 1 μg/mL doxycycline was added for at least 1 day to induce Env expression. Primary cell isolation and culture and HIV-1 isolates used for in vitro cytotoxicity assays are subsequently described.

### Design and Production of Basic and MP3 DARTs

Basic HIVxCD3 DARTs consist of two covalently linked polypeptide chains: Chain 1: CD3VL-HIVVH-ASTKG-E-coil, and Chain 2: HIVVL-CD3VH-ASTKG-K-coil. The oppositely charged E/K-coil domain [95], located at the carboxyl terminus of each chain and containing an interchain disulfide bond, drives heterodimer formation. HIVxCD3 MP3 DARTs consist of three covalently linked polypeptide chains: Chain 1: CD3VL-HIVVH-ASTKG-E-coil-Fc, Chain 2: HIVVL-CD3VH-ASTKG-K-coil, and Chain 3: Fc. The Fc (human IgG1) sequence was modified by point mutations (L234A/L235A) to greatly reduce or abolish binding to activating FcγRs and complement [56]. Chains 1 and 2 form a heterodimer by virtue of the E/K-coil dimerization domain and interchain disulfide bond. Chains 1 and 3 are covalently linked by two disulfide bonds in the Fc hinge region. HIV arms were based on the VH and VL sequences of the following anti-Env mAbs: A32, 7B2, PGT121, PGT145, VRC01, and 10E8 (GenBank accession numbers listed at the end of the Materials and Methods). The CD3 arm was derived from hXR32, a humanized mouse anti-human CD3ε mAb, which cross-reacts with CD3ε from cynomolgus and rhesus macaques [28,57]. A control DART was similarly constructed by replacing the HIV arm with an irrelevant specificity from palivizumab (an anti-RSV mAb) [42].

For the basic DARTs, Chain 1 and Chain 2 coding sequences were cloned into the bicistronic CET1019AD UCOE vectors (EMD Millipore), transfected into CHO cells to generate stable cell lines, and the basic DART proteins were purified as described previously [29]. For the MP3 DARTs, Chain 1 and Chain 2 coding sequences were cloned into a modified CET1019AD vector that contains a neomycin resistance gene, and Chain 3 sequence into the monocistronic CET1019AS UCOE vector. The two plasmids were co-transfected into CHO cells for the generation of stable cell lines. The MP3 DART proteins were purified by affinity chromatography using Protein A Sepharose and followed by SEC when necessary. Approximate size and homogeneity of purified DART proteins in basic or MP3 format were analyzed by SDS-PAGE (NuPAGE Bis-Tris gel system; Invitrogen) and analytical SEC (TSK GS3000SWxL SE-HPLC; Tosoh Bioscience).

### Antigen Binding

DART binding to antigen proteins was measured by ELISA. For monospecific binding assays, a MaxiSorp microtiter plate (Nunc) coated with recombinant protein (human CD3ε/δ heterodimer or JR-FL gp140ΔCF) in bicarbonate buffer was blocked with 3% BSA and 0.1% Tween-20. DART proteins were applied, followed by sequential addition of biotinylated anti-EK coil antibody (recognizes the E/K heterodimerization region of DART proteins) and streptavidin-HRP (BD Biosciences). For bi-specific DART binding assays, the plate was coated with JRFL gp140ΔCF, and DART application was followed by sequential addition of biotinylated CD3ε/δ and streptavidin-HRP. For binding assays with anti-Env IgGs, the plate was coated with JR-FL gp140ΔCF, and IgG application was followed by sequential addition of biotinylated anti-human IgG1 Fc antibody and streptavidin-HRP. HRP activity was detected with SuperSignal ELISA Pico chemiluminescent substrate (Thermo Scientific).

### Cell Surface Env Binding

DART binding to cell lines expressing HIV-1 Env was measured by flow cytometry. DARTs at 4 μg/mL were incubated with 10^5^ cells in 200 μL FACS buffer containing 10% human AB serum for 30 minutes at room temperature. After washing, cells were resuspended in 100 μL of 1 μg/mL biotin-conjugated mouse anti-EK antibody, mixed with 1:500 diluted streptavidin-PE and incubated in the dark for 45 minutes at 2–8°C. Cells were washed, resuspended with FACS buffer, and analyzed by flow cytometry using a FACSCalibur (BD Biosciences) and FlowJo software (TreeStar).

### In Vitro HIV-Infected Cell Killing Assay

PBMCs from healthy participants (AllCells) were isolated by Ficoll-plaque gradient. Total CD4 T cells were isolated from PBMCs using an EasySep Human CD4+ T cell Enrichment Kit (Stemcell Technologies). CD4 T cells (5 × 10^7^) were infected with HIV-1 strain BaL or with the HIV-1 clinical isolates HIV 92/RW/008 or HIV IN/93/905 (NIH AIDS Reagent Program). Infection was done with 50–100 ng p24/million CD4 T cells by spinfecting at 1200 ×*g* for 2 hours [49,50]. Cells were incubated for 5 days at 37°C in RPMI plus 10% FBS with 30 U/mL IL-2 (Invitrogen). PBMCs drawn and isolated on the same day were frozen in 90% heat-inactivated FBS and 10% DMSO. PBMCs were thawed 1 day prior to co-culture, and cells were rested overnight in media at 37°C. On the day of co-culture, CD8 T cells were isolated from thawed PBMCs using an EasySep Human CD8+ T cell Enrichment Kit (Stemcell Technologies). CD8 T cells were co-cultured with infected CD4 T cells at a CD8 T cell:CD4 T cell ratio of 2:1 with varying concentrations of DARTs for 3 days at 37°C.

#### Cell-associated p24

FACS was used to measure reduction in HIV protein, as previously described [48]. After incubation, cells were stained with live/dead Fixable Aqua Dead Cell Stain (Invitrogen), then with PE-Cy7-labeled antibody to CD4 (BD Biosciences) and APC-H7-labeled antibody to CD8 (BD Biosciences). Cells were fixed and permeabilized with Intracellular Fixation & Permeabilization Buffer Set (eBioscience), stained with PE labeled antibody to Gag p24 (Beckman Coulter), and analyzed by flow cytometry using a LSRFortessa (BD Biosciences) and FlowJo software (TreeStar).

#### Cell-associated vRNA

After 3 days of co-culture, plates were spun at 500 ×*g* for 5 min, culture media discarded and the cell pellets were resuspended in RLT buffer containing B2M and were stored at -80°C until RNA isolation. Total RNA was isolated using the RNeasy 96 kit (Qiagen) and cell-associated vRNA levels in these samples were analyzed by a robotic COBAS Ampliprep/Taqman system (Roche Diagnostics), which extracts total nucleic acid and quantifies HIV RNA in copies per milliliter using the HIV Test, v2.0 kit (Roche Diagnostics). RNA from three individual wells was tested for each experimental condition and for testing purpose each RNA sample was diluted 1 to 2,000 in molecular grade water in order get vRNA levels within the detection range of the Cobas system. Maximum vRNA copies/mL detected in these samples ranged from 30,000 to 60,000 for IN and BaL infected samples respectively. Final data is represented as % reduction in vRNA levels in DART treated co-cultures with comparison to No DART treated co cultures.

#### Cell-Associated vDNA

Culture plates were spun at 500 ×*g* for 5 min, culture media discarded and the cell pellets were directly stored at -80°C until DNA isolation. DNA was isolated using the EZ1 DNA tissue kit (Qiagen). Quantitative PCR for total cell associated HIV-DNA was performed using the ViiA 7 Real-Time PCR system and Taqman fast advanced master mix (Life Technologies) in a final volume of 25 μL per reaction using 400 nM primers and 200 nM probe and 100 ng of DNA. Primers and probe set against CCR5 gene (2 copies/genome) were used as internal control standard to account for input DNA quality. DNA from three replicate wells was tested for each experimental condition. For HIV specific q-PCR, primers and probe set used were from HIV integrase: INT-Fw: (5’-TTTGGAAAGGACCAGCAAA-3’), INT-Rv: 5’-(CCTGCCATCTGTTTTCCA-3’) and INT-probe: 5’-[6FAM]-AAAGGTGAAGGGGCAGTAGTAATACA-[TAMRA]-3’. To generate HIV specific standard curve U1 cells containing 2 integrated HIV DNA copies/cell were used. 10-fold serial dilution of the U1 DNA ranging from 20,000 to 2 HIV copies/well were used for the generation of the standard curve. DNA isolated from PBMCs from healthy participants was spiked in with U1 standard curve to make DNA concentration to 100 ng at each dilution point. Data is represented as % reduction in vDNA levels in DART treated co-cultures with comparison to no DART treated cultures.

### Ex Vivo HIV Reservoir Assays

PBMCs were isolated by Ficoll-plaque gradient from leukapheresis samples of HIV-infected participants treated with cART and resuspended at 3 million cells/ml in RPMI with 10% FBS, penicillin/streptomycin, and 100 nM elvitegravir/100 nM efavirenz to prevent new rounds of infection. For experiments with unstimulated cells, combination of 200 pM PGT121xCD3 and 200 pM 7B2xCD3 DART or 400 pM RSVxCD3 alone (negative control) DART were added to cells and incubated in a 37°C, 5% CO2 incubator for 14 days. Every 3 to 4 days, media was removed and added back with the appropriate DARTs and antivirals. For experiments with stimulated cells, a combination of 200 pM PGT121xCD3 and 200 pM 7B2xCD3 DART or 400 pM RSVxCD3 alone (negative control) DART were added together with 1 μM indolactam, and cultures were incubated in a 37°C, 5% CO2 incubator for 7 days. Total CD4 T cells were then purified by negative selection from each culture using an EasySep Human CD4+ T cell Enrichment Kit (Stemcell Technologies). CD4 T cells were plated in 24-well plates at 1 million cells/mL in 2 mL of media containing 100 nM elvitegravir and 100 nM efavirenz and incubated for 3 days. To measure HIV RNA levels, plates were spun at 500 ×*g* for 5 min, and 1 mL of culture supernatant was analyzed by a robotic COBAS Ampliprep/Taqman system (Roche Diagnostics), which extracts total nucleic acid and quantifies HIV RNA in copies per milliliter using the HIV Test, v2.0 kit (Roche Diagnostics).

### In Vitro HIV Spreading Assay in Unstimulated CD4 Cells

Total CD4 T cells from healthy participants’ PBMCs were purified by negative selection using EasySep magnetic beads (Stemcell Technologies). 50×10^6^ CD4 T cells were infected with 50 ng-100 ng p24/ml of lab strain BaL by spinfecting at 1200 ×*g* for 2 hours [48,49]. Cells were washed twice post spinfection and incubated at 37°C with 30 U/mL IL-2 (Invitrogen). MP3 DARTs were added 24 hours post spinfection at varying concentrations. Cells were stained 72 hours post addition of DARTs with live/dead Fixable Aqua Dead Cell Stain (Invitrogen), then with APC-labeled antibody to CD25 (BD Biosciences), PE-labeled antibody to CD69 (BD Biosciences), and v450-labeled antibody to HLA-DR (BD Biosciences). Cells were fixed and permeabilized with Intracellular Fixation & Permeabilization Buffer Set (eBioscience), stained with PE labeled antibody to Gag p24 (Beckman Coulter), and analyzed by flow cytometry using a LSRFortessa (BD Biosciences) and FlowJo software (TreeStar).

### In Vitro HIV Replication Assay in Activated CD4 Cells

Total CD4 T cells were isolated from healthy participants’ PBMCs using EasySep Human CD4+ T cell Enrichment Kit. Cells were divided into 2 aliquots post isolation, where one half of the cells were blasted with 5 μg/mL PHA (Sigma Aldrich) and 100 U/mL IL-2 (Invitrogen) at 2x10^6^ cells/mL for 3 days at 37°C. The other half remained in culture with 30 U/mL IL-2 at 37°C. Three days post activation, cells were infected with 50–100 ng p24/million cells of lab strain BaL by spinfecting at 1200 × g for 2 hours [48,49]. Cells were washed twice post spinfection. The unstimulated portion of cells was labeled with Cell Trace CFSE (Invitrogen) on day 5 and co-cultured with infected blasted cells at 1:1 ratio with or without MP3 DARTs. Cells were stained 48 hours post addition of DARTs with live/dead Fixable Aqua Dead Cell Stain, then with APC-labeled antibody to CD25, PE-labeled antibody to CD69, and v450-labeled antibody to HLADR. Cells were fixed and permeabilized with Intracellular Fixation & Permeabilization Buffer Set (eBioscience), stained with PE labeled antibody to Gag p24 (Beckman Coulter), and analyzed by flow cytometry using a LSRFortessa (BD Biosciences) and FlowJo software (TreeStar).

### Pharmacokinetics (PK) in Human FcRn Transgenic Mice

Female mice (strain B6.Cg-*Fcgrt* ^*tm1Dcr*^ Tg(FCGRT)276Dcr; Jackson Laboratories) were injected intravenously with A32xCD3 MP3 DART diluted in phosphate-buffered saline at a dose level of 5 mg/kg (total of 6 animals). Blood samples for serum were collected from subgroups of 3 animals per time point over a period of 21 days. Concentrations of DART in serum were quantitatively measured by ELISA with immobilized goat anti-hXR32 antibody, which recognizes the anti-CD3 domain (hXR32) of the DART, for capture and goat anti-human IgG Fc-biotin together with streptavidin-horseradish peroxidase (SA-HRP) for detection. The pharmacokinetic parameters were determined using WinNonlin software (Pharsight).

### Accession Numbers (GenBank, NCBI) for the Anti-Env CDRs

A32 [3TNM_H, 3TNM_L]; 7B2 [AFQ31502, AFQ31503], PGT121 [JN201894.1, JN201911.1], PGT145 [JN201910.1, JN201927.1], VRC01 [GU980702.1, GU980703.1] and 10E8 [JX645769.1, JX645770.1].

## Supporting Information

S1 Fig Cell surface binding. (A) HIVxCD3 DART binding to Env-transfected cells. Histograms represent the relative levels of HIVxCD3 DART binding to HEK293-D371 cells expressing CM244 subtype AE *Env* (upper panels) and HEK293-D375 cells expressing subtype AE 92Th023 *Env* (lower panels) in the absence (blue lines) or presence (red lines) of doxycycline (dox), which induces Env expression. (B) Parental anti-HIV Env IgG binding to HIV-infected cells. Unstimulated CD4 T cells were mock-infected or infected with HIV-1 BaL. After 5 days in culture, cells were stained with anti-p24 and biotinylated forms of the indicated bNAbs prior to FACS analysis.
(PDF)

S2 Fig In vitro CD8 T cell-dependent cytolysis of HIV-infected CD4 T cells mediated by HIVxCD3 DARTs. Unstimulated primary CD4 T cells were infected with HIV in vitro for 6 days, as previously described. Autologous CD8 T cells were cultured with HIV-infected CD4 T cells at a 2:1 CD8:CD4 ratio in the absence or presence of control DART (RSVxCD3) or active DART (HIVxCD3). After 72 hours of co-culture, cells were harvested, and were first stained with Live dead aqua dye before surface staining with anti-CD4 and anti-CD8 Abs. Surface stained cells were perm-fixed, and stained with anti-p24 Ab. Representative data for the PGT121xCD3-redirected CD8 T cell activity against cells from a participant infected with HIV-1 BaL are depicted. The percent reductions of HIV-infected p24+ CD4+ T cells were calculated for each condition relative to the no DART control, as indicated. Equal volumes and a minimum of 200,000 cells were analyzed for all groups. Variations in the numbers of uninfected p24- CD4+ T cells were <2,000 (<1%) between groups, indicating specific reduction of HIV-infected cells by HIVxCD3 DART.
(PDF)

S3 Fig Optimal HIVxCD3 DART-dependent killing of HIV-infected CD4 T cells was achieved at a CD8:CD4 T cell ratio of 2:1 with a co-culture period of 72 hours. Unstimulated CD4 T cells were infected with HIV-1 BaL and co-cultured with autologous CD8 T cells at the indicated CD8:CD4 T cell ratios and with 150 pM DARTs for the times indicated. Cytotoxicity values were determined by FACS, as described in Materials and Methods. Representative data from a single participant is depicted.
(PDF)

S4 Fig HIVxCD3 DARTs reduce cell-associated HIV p24 protein, HIV RNA, and integrated HIV DNA. Unstimulated CD4 T cells were infected with HIV-1 BaL for 6 days and co-cultured with autologous CD8 T cells at a CD8:CD4 T cell ratio of 2:1 in the absence or presence of PGT121xCD3 + 7B2xCD3 HIV DARTs at 200 pM each or RSVxCD3 control DART at 400 pM. After 72 hours of co-culture, cells were analyzed by: FACS for intracellular p24 protein expression, qRT-PCR (COBAS) for vRNA, and qPCR for total vDNA, as described in the Methods. The average of data from 2 participants are depicted.
(PDF)

S5 Fig Stability of HIVxCD3 DARTs in the presence of resting or activated CD4^+^ T cells in vitro. Selected HIVxCD3 DARTs in basic or MP3 format at a concentration of 50 ng/mL were incubated in (A) culture media (complete RPMI, 10% FCS, in (B) culture media plus 1 x 10^6^ CD4+ T cells, or in (C) culture media, 1 x 10^6^ CD4+ T cells, 50 ng/mL PMA and 500 ng/mL ionomycin for 5 days at 37°C. Samples were collected on the days indicated, and DART concentrations were measured by quantitative ELISA. % recovery on each daywas determined compared to day 0.
(PDF)

S6 Fig Neutralization activity of parental anti-HIV Env IgGs. The three HIV isolates indicated were pre-incubated with the bNAbs PGT121, PGT145, VRC01, or 10E8 prior to infection of CEM-CCR5 reporter cells using luciferase as a read-out in a standard single cycle neutralization assay. For HIV-1 BaL and RW strains, an isotype control Ab TCON0 was also included. The IC50s in μg/ml for each bNAb and HIV isolate are indicated. Representative results from a single participant are depicted.
(PDF)

S7 Fig Schematics of the ex vivo models for the testing of HIV DARTs. PBMCs from HIV-infected participants on suppressive cART were either unstimulated (Resting model) or stimulated with 1uM indolactam (PKC-activated model) in the absence or presence of active (HIVxCD3) or control (RSVxCD3) DARTs. In the resting model, supernatant vRNA was quantitated after 8 days and 14 days in culture. In the PKC-activated model, total CD4 T cells were purified after 7 days and re-stimulated with 1 μM indolactam for an additional 3 days prior to vRNA quantitation.
(PDF)

S8 Fig The PKC agonist indolactam is an effective HIV latency reversal agent ex vivo. (A) PBMCs from 8 HIV-infected participants on suppressive cART were treated with DMSO control or with 1 μM indolactam, a PKC agonist. After 7 days of culture, supernatant HIV RNA was quantitated by COBAS qRT-PCR (*p<0.05 two-tailed student’s t-test). Horizontal bars represent the medians for each group. (B) PBMCs from 4 HIV-infected participants on suppressive cART were cultured untreated or treated with 1 μM indolactam, a PKC agonist, and with or without an HIVxCD3 DART combination (200 pM PGT121xCD3 + 200 pM 7B2xCD3) or control RSVxCD3 DART (400 pM). After 7 days of incubation, total CD4 T cells were isolated from PBMCs and re-stimulated with 1 μM indolactam. After an additional 3 days of incubation, supernatant HIV RNA was quantitated. The No PKC, No DART group was initially treated with DMSO and was re-stimulated with indolactam. All other groups were stimulated and re-stimulated with indolactam. In 4 out of 4 participants in the No DART group, initial stimulation with indolactam alone was sufficient to reduce vRNA after subsequent re-stimulation with indolactam compared to the No PKC, No DART group. These reductions reached statistical significance in 3 out of 4 participants (*p<0.005, 2-tailed Mann-Whitney U-Test).
(PDF)

S9 Fig Binding of HIVxCD3 DARTs in basic and MP3 format. A-C.) Antigen binding by ELISA. Binding of basic DARTs (closed symbols) and MP3 DARTs (open symbols) to (A) human CD3 protein, to (B) JRFL gp140 protein, or to (C) both human CD3 and JRFL gp140 protein. D-E.) Cell surface Env binding by flow cytometry. Histograms represent the levels of (D) basic DARTs or (E) MP3 DARTs bound to HEK293-D371 cells induced by doxycycline to express CM244 subtype AE *Env*. The mean fluorescent intensity (MFI) for controls were 6.4 (unstained, red lines) and 11.0 (no DART). MFI for basic DARTs were 16.6 (RSVxCD3), 65.5 (PGT121xCD3), 269 (PGT145xCD3), 1546 (A32xCD3) and 2633 (7B2xCD3). MFI for MP3 DARTs were 16.7 (RSVxCD3 MP3), 17.0 (PGT121xCD3 MP3), 186 (PGT145xCD3 MP3), 1158 (A32xCD3 MP3) and 1965 (7B2xCD3 MP3).
(PDF)

S10 Fig HIVxCD3 MP3 DARTs do not increase HIV spread during productive infection. (A) CD4 T cell Activation. PGT121xCD3 MP3 or RSVxCD3 MP3 DARTs were added to unstimulated primary CD4 T cells 24 hours post spinfection with HIV BaL. After 5 days of culture, expression of the cell surface activation markers CD25, CD69, and HLA-DR were measured on the uninfected (p24-) and the HIV-infected (p24+) fractions of CD4 T cells by FACS. (B) Activated HIV Infection Model. Primary CD4 T cells were activated by PMA plus ionomycin, infected with HIV (BaL) and incubated in the presence of HIVxCD3 or control MP3 DARTs. A32xCD3 MP3, derived from a non-neutralizing antibody, did not enhance HIV infection relative to RSVxCD3 MP3, while PGT121xCD3 MP3, derived from a broadly neutralizing antibody reduced the HIV infection. (C) Resting HIV Infection Model. Unstimulated primary CD4 T cells were infected with HIV (BaL) and incubated in the presence of HIVxCD3 or control MP3 DARTs. PGT121xCD3 MP3 did not enhance HIV infection relative to RSVxCD3 MP3.
(PDF)
