# Supplementary material for: Targeting HIV Reservoir in Infected CD4 T Cells by Dual-Affinity Re-targeting Molecules (DARTs) that Bind HIV Envelope and Recruit Cytotoxic T Cells
Source: PLoS Pathog. 2015 Nov 5;11(11):e1005233. doi: 10.1371/journal.ppat.1005233 (PMC4634948; doi:10.1371/journal.ppat.1005233)
Supplement: S1 Fig — (A) HIVxCD3 DART binding to Env-transfected cells. Histograms represent the relative levels of HIVxCD3 DART binding to HEK293-D371 cells expressing CM244 subtype AE Env (upper panels) and HEK293-D375 cells expressing subtype AE 92Th023 Env (lower panels) in the absence (blue lines) or presence (red lines) of doxycycline (dox), which induces Env expression. (B) Parental anti-HIV Env IgG binding to HIV-infected cells. Unstimulated CD4 T cells were mock-infected or infected with HIV-1 BaL. After 5 days in culture, cells were stained with anti-p24 and biotinylated forms of the indicated bNAbs prior to FACS analysis. (PDF) [file ppat.1005233.s001.pdf]

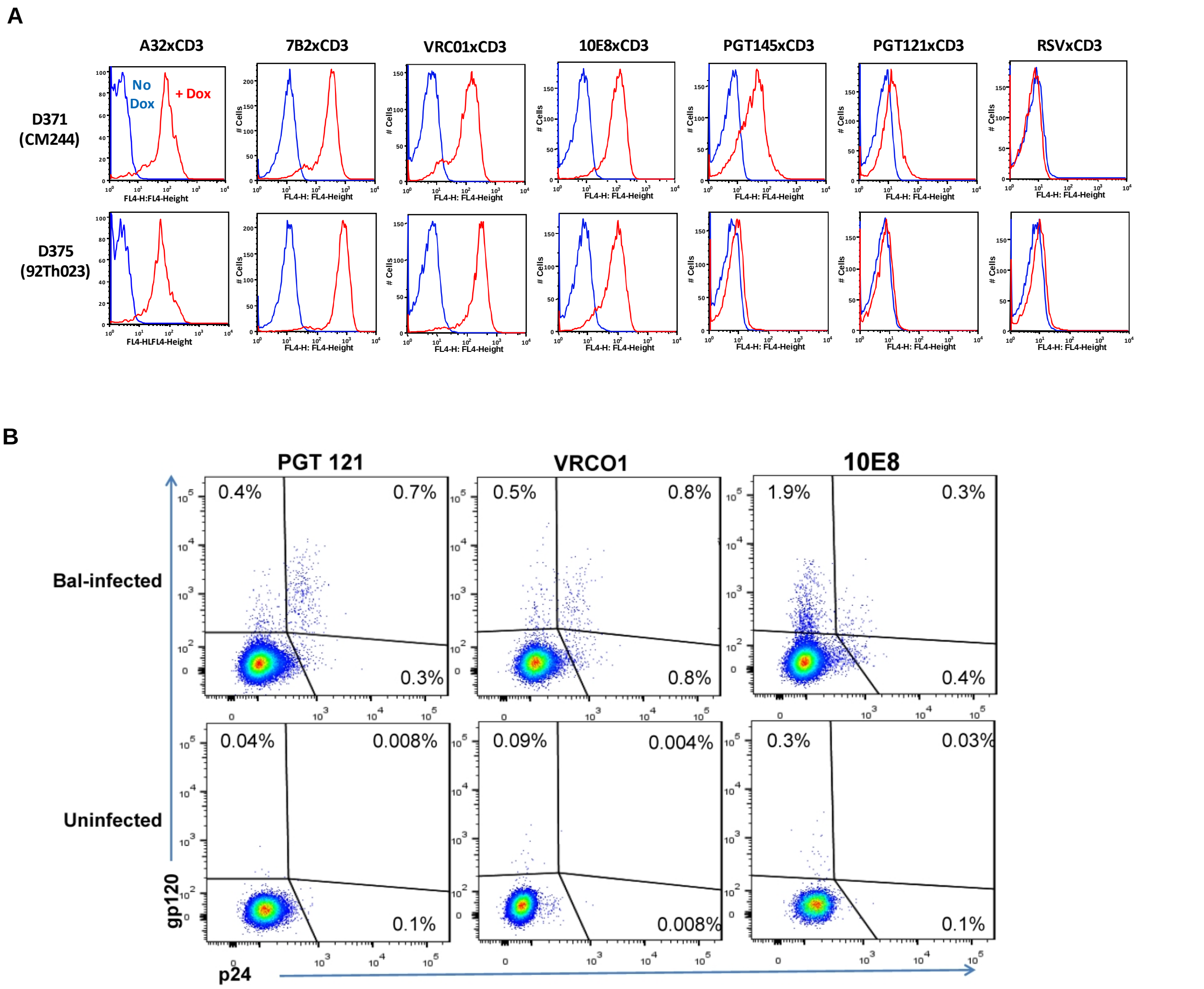

Supplementary Fig. 1. Cell surface binding. (A) HIVxCD3 DART binding to Env-transfected cells. Histograms represent the relative levels of HIVxCD3 DART binding to HEK293-D371 cells expressing CM244 subtype AE *Env* (upper panels) and HEK293-D375 cells expressing subtype AE 92Th023 *Env* (lower panels) in the absence (blue lines) or presence (red lines) of doxycycline (dox), which induces Env expression. **(B) Parental anti-HIV Env IgG binding to HIV-infected cells.** Unstimulated CD4 T cells were mock-infected or infected with HIV-1 BaL. After 5 days in culture, cells were stained with anti-p24 and biotinylated forms of the indicated bNAbs prior to FACS analysis.
