# Supplementary material for: Targeting HIV Reservoir in Infected CD4 T Cells by Dual-Affinity Re-targeting Molecules (DARTs) that Bind HIV Envelope and Recruit Cytotoxic T Cells
Source: PLoS Pathog. 2015 Nov 5;11(11):e1005233. doi: 10.1371/journal.ppat.1005233 (PMC4634948; doi:10.1371/journal.ppat.1005233)
Supplement: S2 Fig — Unstimulated primary CD4 T cells were infected with HIV in vitro for 6 days, as previously described. Autologous CD8 T cells were cultured with HIV-infected CD4 T cells at a 2:1 CD8:CD4 ratio in the absence or presence of control DART (RSVxCD3) or active DART (HIVxCD3). After 72 hours of co-culture, cells were harvested, and were first stained with Live dead aqua dye before surface staining with anti-CD4 and anti-CD8 Abs. Surface stained cells were perm-fixed, and stained with anti-p24 Ab. Representative data for the PGT121xCD3-redirected CD8 T cell activity against cells from a participant infected with HIV-1 BaL are depicted. The percent reductions of HIV-infected p24+ CD4+ T cells were calculated for each condition relative to the no DART control, as indicated. Equal volumes and a minimum of 200,000 cells were analyzed for all groups. Variations in the numbers of uninfected p24- CD4+ T cells were <2,000 (<1%) between groups, indicating specific reduction of HIV-infected cells by HIVxCD3 DART. (PDF) [file ppat.1005233.s002.pdf]

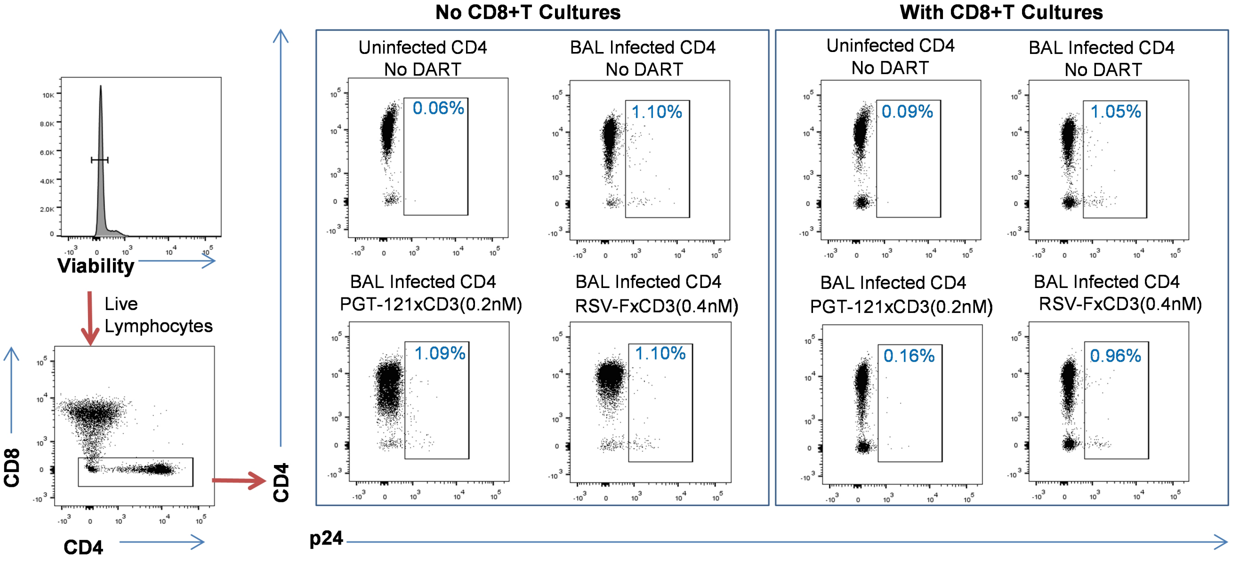

Sample Calculation

% Reduction CD4+p24+ vs . No DART

Active HIV DART PGT121xCD3: ((1.05-0.16)/1.05)*100 = 85%

Control DART RSV-FxCD3: ((1.05-0.96)/1.05)*100 = 8.6%

Supplementary Fig. 2. In vitro CD8 T cell-dependent cytolysis of HIV-infected CD4 T cells mediated by HIVxCD3 DARTs. Unstimulated primary CD4 T cells were infected with HIV in vitro for 6 days, as previously described. Autologous CD8 T cells were cultured with HIV-infected CD4 T cells at a 2:1 CD8:CD4 ratio in the absence or presence of control DART (RSVxCD3) or active DART (HIVxCD3). After 72 hours of co-culture, cells were harvested, and were first stained with Live dead aqua dye before surface staining with anti-CD4 and anti-CD8 Abs. Surface stained cells were perm-fixed, and stained with anti-p24 Ab. Representative data for the PGT121xCD3-redirected CD8 T cell activity against cells from a donor infected with HIV-1 BaL are depicted. The percent reductions of HIV-infected p24+ CD4+ T cells were calculated for each condition relative to the no DART control, as indicated. Equal volumes and a minimum of 200,000 cells were analyzed for all groups. Variations in the numbers of uninfected p24- CD4+ T cells were <2,000 (<1%) between groups, indicating specific reduction of HIV-infected cells by HIVxCD3 DART.
