# Supplementary material for: Targeting HIV Reservoir in Infected CD4 T Cells by Dual-Affinity Re-targeting Molecules (DARTs) that Bind HIV Envelope and Recruit Cytotoxic T Cells
Source: PLoS Pathog. 2015 Nov 5;11(11):e1005233. doi: 10.1371/journal.ppat.1005233 (PMC4634948; doi:10.1371/journal.ppat.1005233)
Supplement: S3 Fig — Unstimulated CD4 T cells were infected with HIV-1 BaL and co-cultured with autologous CD8 T cells at the indicated CD8:CD4 T cell ratios and with 150 pM DARTs for the times indicated. Cytotoxicity values were determined by FACS, as described in Materials and Methods. Representative data from a single participant is depicted. (PDF) [file ppat.1005233.s003.pdf]

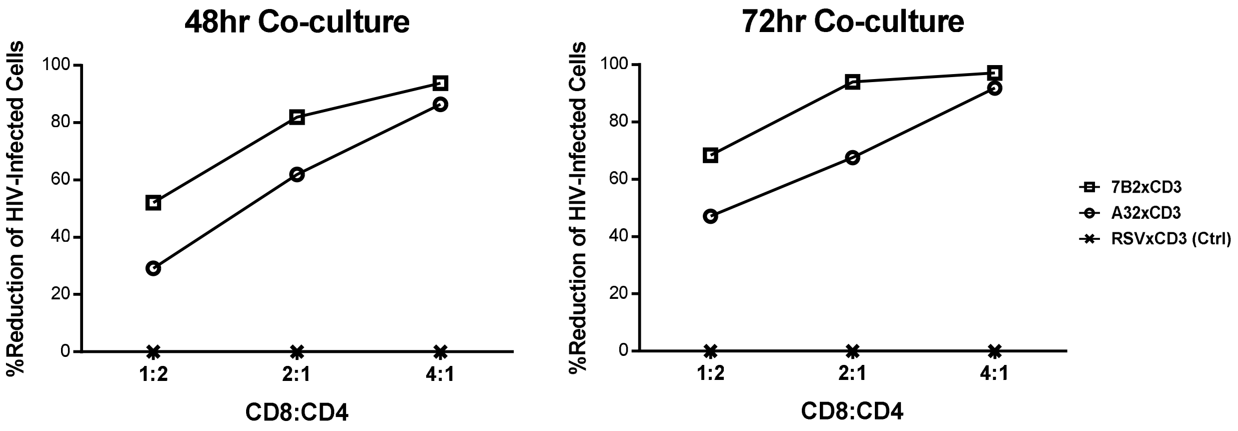

Supplementary Fig. 3. Optimal HIVxCD3 DART-dependent killing of HIV-infected CD4 T cells was achieved at a CD8:CD4 T cell ratio of 2:1 with a co-culture period of 72 hours. Unstimulated CD4 T cells were infected with HIV-1 BaL and co-cultured with autologous CD8 T cells at the indicated CD8:CD4 T cell ratios and with 150 pM DARTs for the times indicated. Cytotoxicity values were determined by FACS, as described in Materials and Methods. Representative data from a single donor is depicted.
