# Supplementary material for: Targeting HIV Reservoir in Infected CD4 T Cells by Dual-Affinity Re-targeting Molecules (DARTs) that Bind HIV Envelope and Recruit Cytotoxic T Cells
Source: PLoS Pathog. 2015 Nov 5;11(11):e1005233. doi: 10.1371/journal.ppat.1005233 (PMC4634948; doi:10.1371/journal.ppat.1005233)
Supplement: S4 Fig — Unstimulated CD4 T cells were infected with HIV-1 BaL for 6 days and co-cultured with autologous CD8 T cells at a CD8:CD4 T cell ratio of 2:1 in the absence or presence of PGT121xCD3 + 7B2xCD3 HIV DARTs at 200 pM each or RSVxCD3 control DART at 400 pM. After 72 hours of co-culture, cells were analyzed by: FACS for intracellular p24 protein expression, qRT-PCR (COBAS) for vRNA, and qPCR for total vDNA, as described in the Methods. The average of data from 2 participants are depicted. (PDF) [file ppat.1005233.s004.pdf]

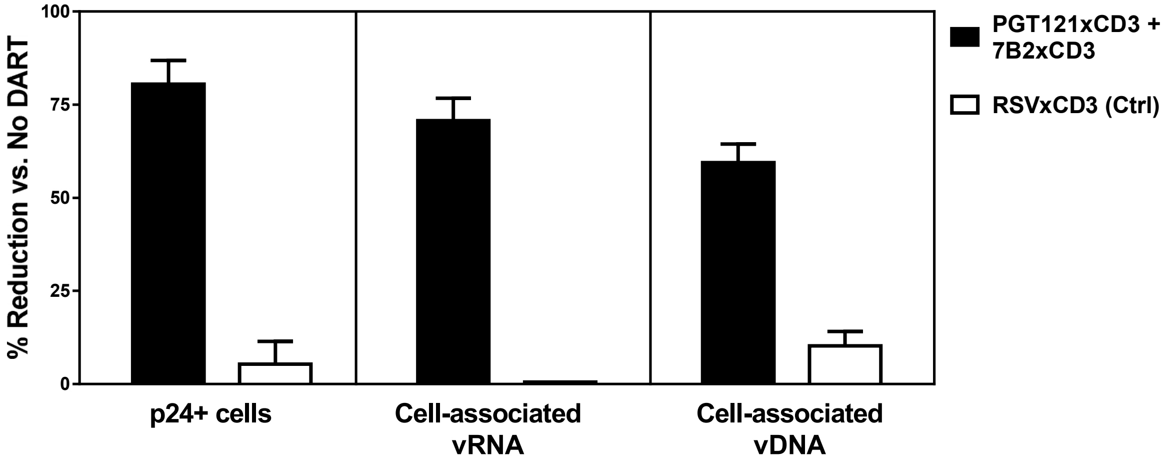

Supplementary Fig. 4. HIVxCD3 DARTs reduce cell-associated HIV p24 protein, HIV RNA, and integrated HIV DNA. PGT121xCD3 + 7B2xCD3 HIV DARTs (200 pM each) were added to unstimulated primary CD4 T cells 24 hours post spinfection with HIV BaL. After 5 days of culture, cells were analyzed: by FACS for intracellular p24 protein expression, by qRT-PCR (COBAS) for vRNA, and by qPCR for total vDNA. The average of data from2 donors are depicted.
