# Supplementary material for: Targeting HIV Reservoir in Infected CD4 T Cells by Dual-Affinity Re-targeting Molecules (DARTs) that Bind HIV Envelope and Recruit Cytotoxic T Cells
Source: PLoS Pathog. 2015 Nov 5;11(11):e1005233. doi: 10.1371/journal.ppat.1005233 (PMC4634948; doi:10.1371/journal.ppat.1005233)
Supplement: S5 Fig — Selected HIVxCD3 DARTs in basic or MP3 format at a concentration of 50 ng/mL were incubated in (A) culture media (complete RPMI, 10% FCS, in (B) culture media plus 1 x 10^6^ CD4+ T cells, or in (C) culture media, 1 x 10^6^ CD4+ T cells, 50 ng/mL PMA and 500 ng/mL ionomycin for 5 days at 37°C. Samples were collected on the days indicated, and DART concentrations were measured by quantitative ELISA. % recovery on each daywas determined compared to day 0. (PDF) [file ppat.1005233.s005.pdf]

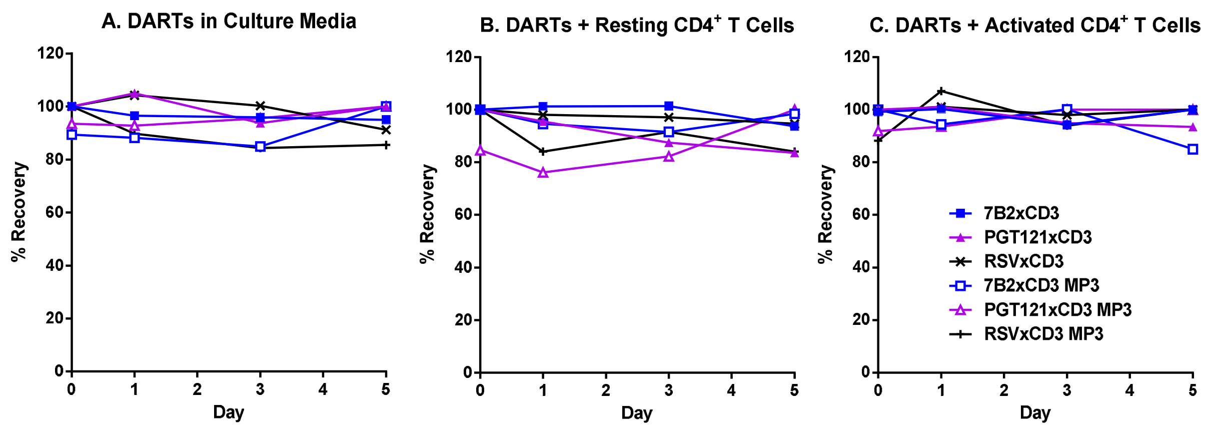

Supplementary Fig. 5. Stability of HIVxCD3 DARTs in the presence of resting or activated $CD4^+$ T cells in vitro. Selected HIVxCD3 DARTs in basic or MP3 format at a concentration of 50 ng/mL were incubated in (A) culture media (complete RPMI, 10% FCS, in (B) culture media plus 1×10^6 CD4+ T cells, or in (C) culture media, 1×10^6 CD4+ T cells, 50 ng/mL PMA and 500 ng/mL ionomycin for 5 days at 37°C. Samples were collected on the days indicated, and DART concentrations were measured by quantitative ELISA. % recovery on each daywas determined compared to day 0.
