# Supplementary material for: Targeting HIV Reservoir in Infected CD4 T Cells by Dual-Affinity Re-targeting Molecules (DARTs) that Bind HIV Envelope and Recruit Cytotoxic T Cells
Source: PLoS Pathog. 2015 Nov 5;11(11):e1005233. doi: 10.1371/journal.ppat.1005233 (PMC4634948; doi:10.1371/journal.ppat.1005233)
Supplement: S6 Fig — The three HIV isolates indicated were pre-incubated with the bNAbs PGT121, PGT145, VRC01, or 10E8 prior to infection of CEM-CCR5 reporter cells using luciferase as a read-out in a standard single cycle neutralization assay. For HIV-1 BaL and RW strains, an isotype control Ab TCON0 was also included. The IC50s in μg/ml for each bNAb and HIV isolate are indicated. Representative results from a single participant are depicted. (PDF) [file ppat.1005233.s006.pdf]

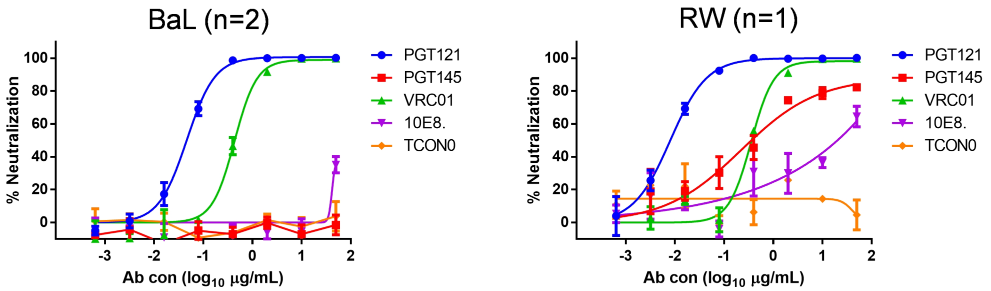

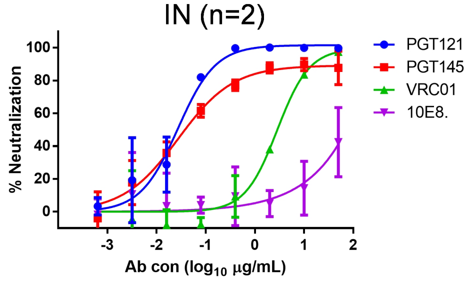

IC50 (ug/mL)	PGT121	PGT145	VRC01	10E8.	TCON0
BaL	0.046	>50	0.438	>50	>50
IN	0.026	0.038	2.851	>50	>50
RW	0.008	0.358	0.362	>50	>50

Supplementary Fig. 6. Neutralization activity of parental anti-HIV Env IgGs. The three HIV isolates indicated were pre-incubated with the bNAbs PGT121, PGT145, VRC01, or 10E8 prior to infection of CEM-CCR5 reporter cells using luciferase as a read-out in a standard single cycle neutralization assay. For HIV-1 BaL and RW strains, an isotype control Ab TCON0 was also included. The IC_{50}s in µg/mL for each bNAb and HIV isolate are indicated. Representative results from a single donor are depicted.
