# Supplementary material for: Targeting HIV Reservoir in Infected CD4 T Cells by Dual-Affinity Re-targeting Molecules (DARTs) that Bind HIV Envelope and Recruit Cytotoxic T Cells
Source: PLoS Pathog. 2015 Nov 5;11(11):e1005233. doi: 10.1371/journal.ppat.1005233 (PMC4634948; doi:10.1371/journal.ppat.1005233)
Supplement: S7 Fig — PBMCs from HIV-infected participants on suppressive cART were either unstimulated (Resting model) or stimulated with 1uM indolactam (PKC-activated model) in the absence or presence of active (HIVxCD3) or control (RSVxCD3) DARTs. In the resting model, supernatant vRNA was quantitated after 8 days and 14 days in culture. In the PKC-activated model, total CD4 T cells were purified after 7 days and re-stimulated with 1 μM indolactam for an additional 3 days prior to vRNA quantitation. (PDF) [file ppat.1005233.s007.pdf]

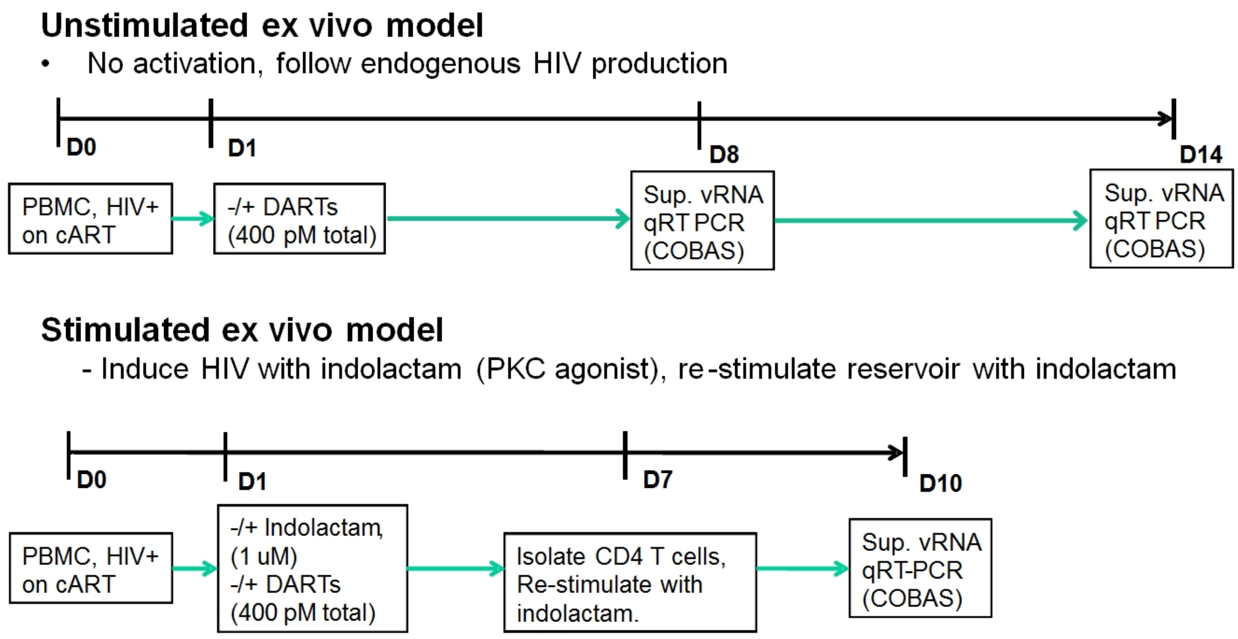

Supplementary Fig. 7. Schematics of the ex vivo models for the testing of HIV DARTs. PBMCs from HIV-infected participants on suppressive cART were either unstimulated (Resting model) or stimulated with 1uM indolactam (PKC-activated model) in the absence or presence of active (HIVxCD3) or control (RSVxCD3) DARTs. In the resting model, supernatant vRNA was quantitated after 8 days and 14 days in culture. In the PKC-activated model, total CD4 T cells were purified after 7 days and re-stimulated with 1 uM indolactam for an additional 3 days prior to vRNA quantitation.
