# Supplementary material for: Targeting HIV Reservoir in Infected CD4 T Cells by Dual-Affinity Re-targeting Molecules (DARTs) that Bind HIV Envelope and Recruit Cytotoxic T Cells
Source: PLoS Pathog. 2015 Nov 5;11(11):e1005233. doi: 10.1371/journal.ppat.1005233 (PMC4634948; doi:10.1371/journal.ppat.1005233)
Supplement: S8 Fig — (A) PBMCs from 8 HIV-infected participants on suppressive cART were treated with DMSO control or with 1 μM indolactam, a PKC agonist. After 7 days of culture, supernatant HIV RNA was quantitated by COBAS qRT-PCR (*p<0.05 two-tailed student’s t-test). Horizontal bars represent the medians for each group. (B) PBMCs from 4 HIV-infected participants on suppressive cART were cultured untreated or treated with 1 μM indolactam, a PKC agonist, and with or without an HIVxCD3 DART combination (200 pM PGT121xCD3 + 200 pM 7B2xCD3) or control RSVxCD3 DART (400 pM). After 7 days of incubation, total CD4 T cells were isolated from PBMCs and re-stimulated with 1 μM indolactam. After an additional 3 days of incubation, supernatant HIV RNA was quantitated. The No PKC, No DART group was initially treated with DMSO and was re-stimulated with indolactam. All other groups were stimulated and re-stimulated with indolactam. In 4 out of 4 participants in the No DART group, initial stimulation with indolactam alone was sufficient to reduce vRNA after subsequent re-stimulation with indolactam compared to the No PKC, No DART group. These reductions reached statistical significance in 3 out of 4 participants (*p<0.005, 2-tailed Mann-Whitney U-Test). (PDF) [file ppat.1005233.s008.pdf]

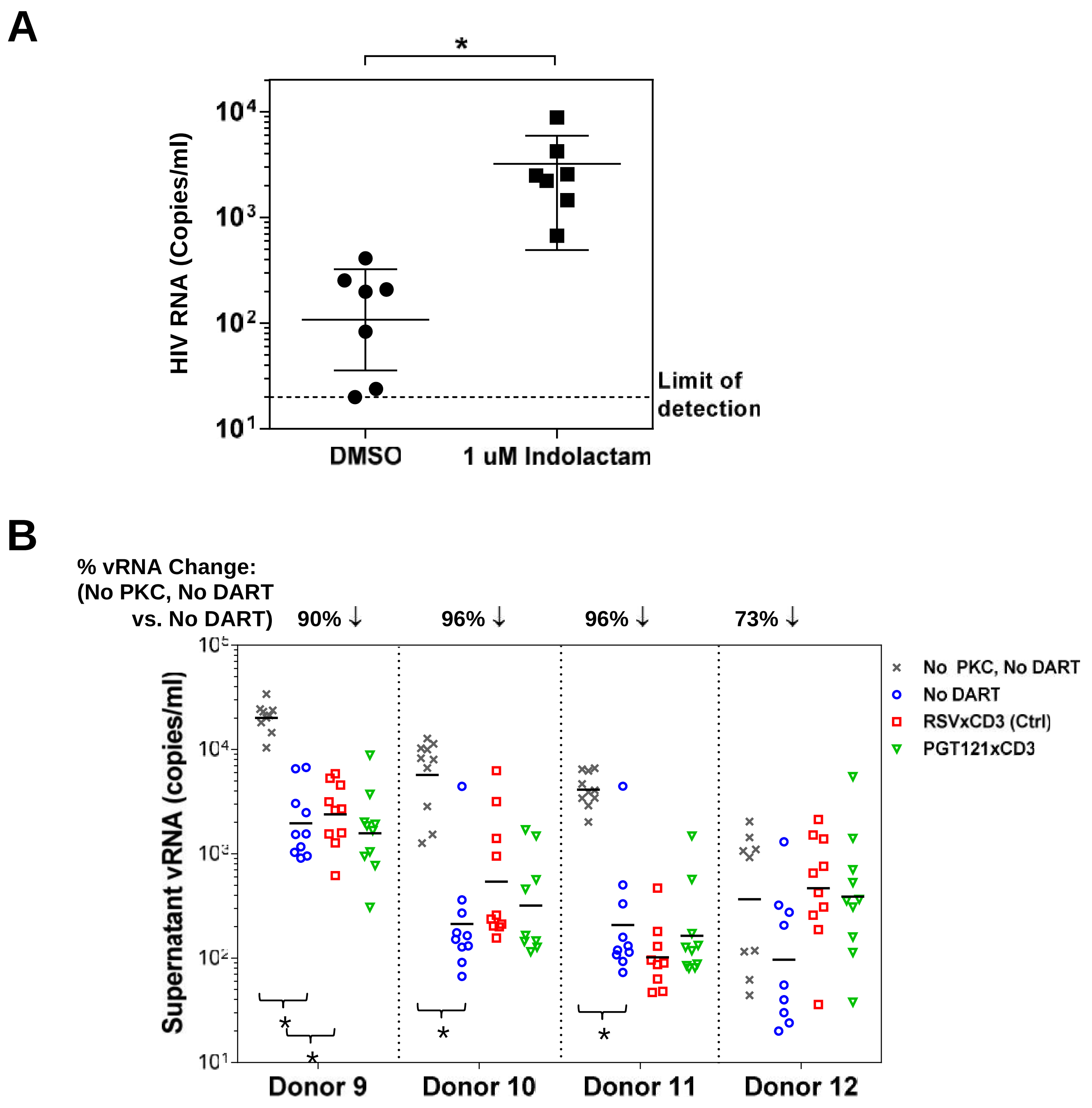

Supplementary Fig. 8. The PKC agonist indolactam is an effective HIV latency reversal agent ex vivo. (A) PBMCs from 8 HIV-infected participants on suppressive cART were treated with DMSO control or with 1 μM indolactam, a PKC agonist. After 7 days of culture, supernatant HIV RNA was quantitated by COBAS qRT-PCR (*p<0.05 two-tailed student's t-test). Horizontal bars represent the medians for each group. **(B)** PBMCs from 4 HIV-infected participants on suppressive cART were cultured untreated or treated with 1 μM indolactam, a PKC agonist, and with or without an HIVxCD3 DART combination (200 pM PGT121xCD3 + 200 pM 7B2xCD3) or control RSVxCD3 DART (400 pM). After 7 days of incubation, total CD4 T cells were isolated from PBMCs and re-stimulated with 1 μM indolactam. After an additional 3 days of incubation, supernatant HIV RNA was quantitated. The No PKC, No DART group was initially treated with DMSO and was re-stimulated with indolactam. All other groups were stimulated and re-stimulated with indolactam. In 4 out of 4 participants in the No DART group, initial stimulation with indolactam alone was sufficient to reduce vRNA after subsequent re-stimulation with indolactam compared to the No PKC, No DART group. These reductions reached statistical significance in 3 out of 4 participants (*p<0.005, 2-tailed Mann-Whitney U-Test).
