# Supplementary material for: Targeting HIV Reservoir in Infected CD4 T Cells by Dual-Affinity Re-targeting Molecules (DARTs) that Bind HIV Envelope and Recruit Cytotoxic T Cells
Source: PLoS Pathog. 2015 Nov 5;11(11):e1005233. doi: 10.1371/journal.ppat.1005233 (PMC4634948; doi:10.1371/journal.ppat.1005233)
Supplement: S9 Fig — A-C.) Antigen binding by ELISA. Binding of basic DARTs (closed symbols) and MP3 DARTs (open symbols) to (A) human CD3 protein, to (B) JRFL gp140 protein, or to (C) both human CD3 and JRFL gp140 protein. D-E.) Cell surface Env binding by flow cytometry. Histograms represent the levels of (D) basic DARTs or (E) MP3 DARTs bound to HEK293-D371 cells induced by doxycycline to express CM244 subtype AE Env. The mean fluorescent intensity (MFI) for controls were 6.4 (unstained, red lines) and 11.0 (no DART). MFI for basic DARTs were 16.6 (RSVxCD3), 65.5 (PGT121xCD3), 269 (PGT145xCD3), 1546 (A32xCD3) and 2633 (7B2xCD3). MFI for MP3 DARTs were 16.7 (RSVxCD3 MP3), 17.0 (PGT121xCD3 MP3), 186 (PGT145xCD3 MP3), 1158 (A32xCD3 MP3) and 1965 (7B2xCD3 MP3). (PDF) [file ppat.1005233.s009.pdf]

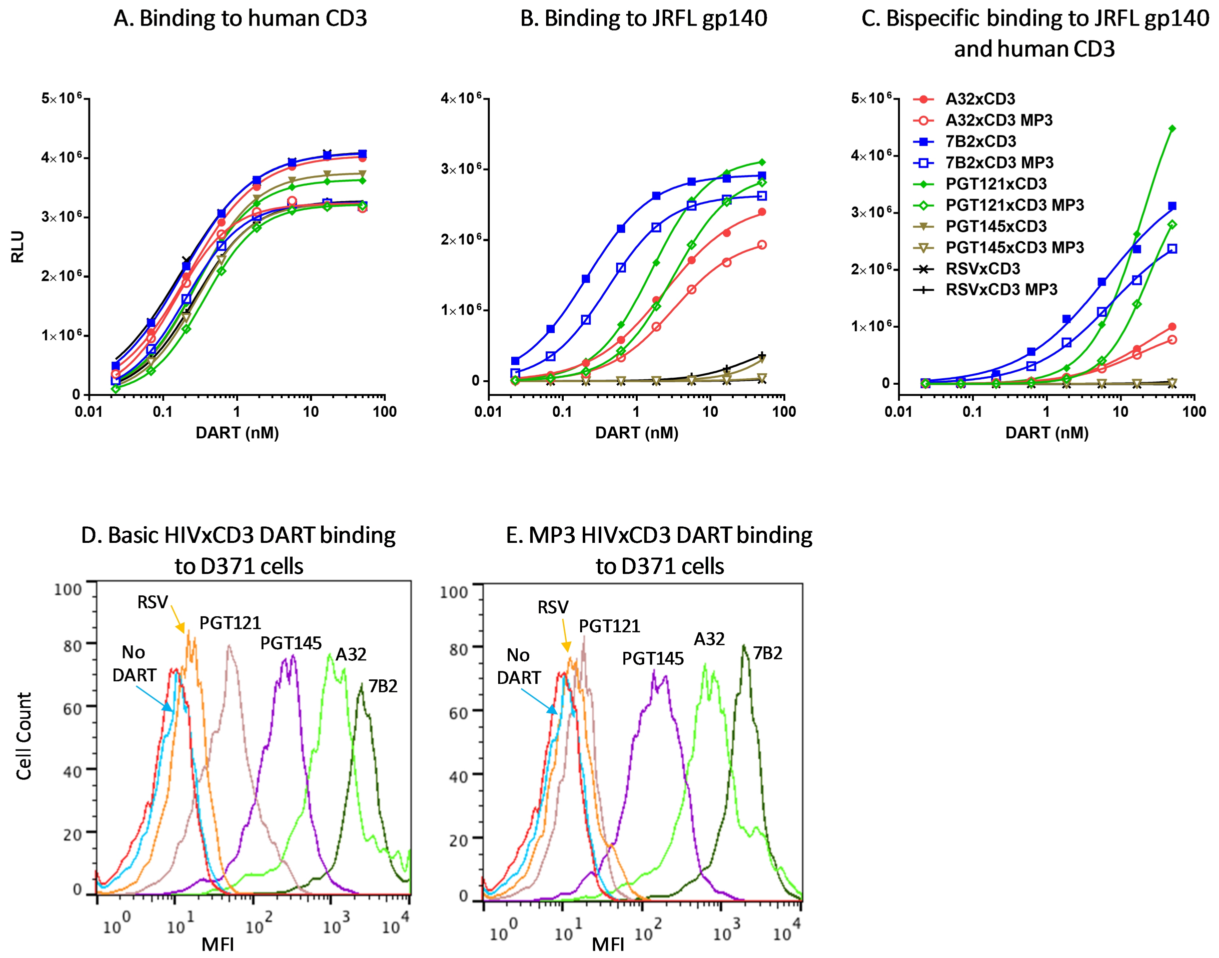

Supplementary Fig. 9. Binding of HIVxCD3 DARTs in basic and MP3 format. A-C.) Antigen binding by ELISA. Binding of basic DARTs (closed symbols) and MP3 DARTs (open symbols) to (A) human CD3 protein, to (B) JRFL gp140 protein, or to (C) both human CD3 and JRFL gp140 protein. **D-E.) Cell surface Env binding by flow cytometry.** Histograms represent the levels of (D) basic DARTs or (E) MP3 DARTs bound to HEK293-D371 cells induced by doxycycline to express CM244 subtype AE *Env*. The mean fluorescent intensity (MFI) for controls were 6.4 (unstained, red lines) and 11.0 (no DART). MFI for basic DARTs were 16.6 (RSVxCD3), 65.5 (PGT121xCD3), 269 (PGT145xCD3), 1546 (A32xCD3) and 2633 (7B2xCD3). MFI for MP3 DARTs were 16.7 (RSVxCD3 MP3), 17.0 (PGT121xCD3 MP3), 186 (PGT145xCD3 MP3), 1158 (A32xCD3 MP3), and 1965 (7B2xCD3 MP3).
