# Supplementary material for: Targeting HIV Reservoir in Infected CD4 T Cells by Dual-Affinity Re-targeting Molecules (DARTs) that Bind HIV Envelope and Recruit Cytotoxic T Cells
Source: PLoS Pathog. 2015 Nov 5;11(11):e1005233. doi: 10.1371/journal.ppat.1005233 (PMC4634948; doi:10.1371/journal.ppat.1005233)
Supplement: S10 Fig — (A) CD4 T cell Activation. PGT121xCD3 MP3 or RSVxCD3 MP3 DARTs were added to unstimulated primary CD4 T cells 24 hours post spinfection with HIV BaL. After 5 days of culture, expression of the cell surface activation markers CD25, CD69, and HLA-DR were measured on the uninfected (p24-) and the HIV-infected (p24+) fractions of CD4 T cells by FACS. (B) Activated HIV Infection Model. Primary CD4 T cells were activated by PMA plus ionomycin, infected with HIV (BaL) and incubated in the presence of HIVxCD3 or control MP3 DARTs. A32xCD3 MP3, derived from a non-neutralizing antibody, did not enhance HIV infection relative to RSVxCD3 MP3, while PGT121xCD3 MP3, derived from a broadly neutralizing antibody reduced the HIV infection. (C) Resting HIV Infection Model. Unstimulated primary CD4 T cells were infected with HIV (BaL) and incubated in the presence of HIVxCD3 or control MP3 DARTs. PGT121xCD3 MP3 did not enhance HIV infection relative to RSVxCD3 MP3. (PDF) [file ppat.1005233.s010.pdf]

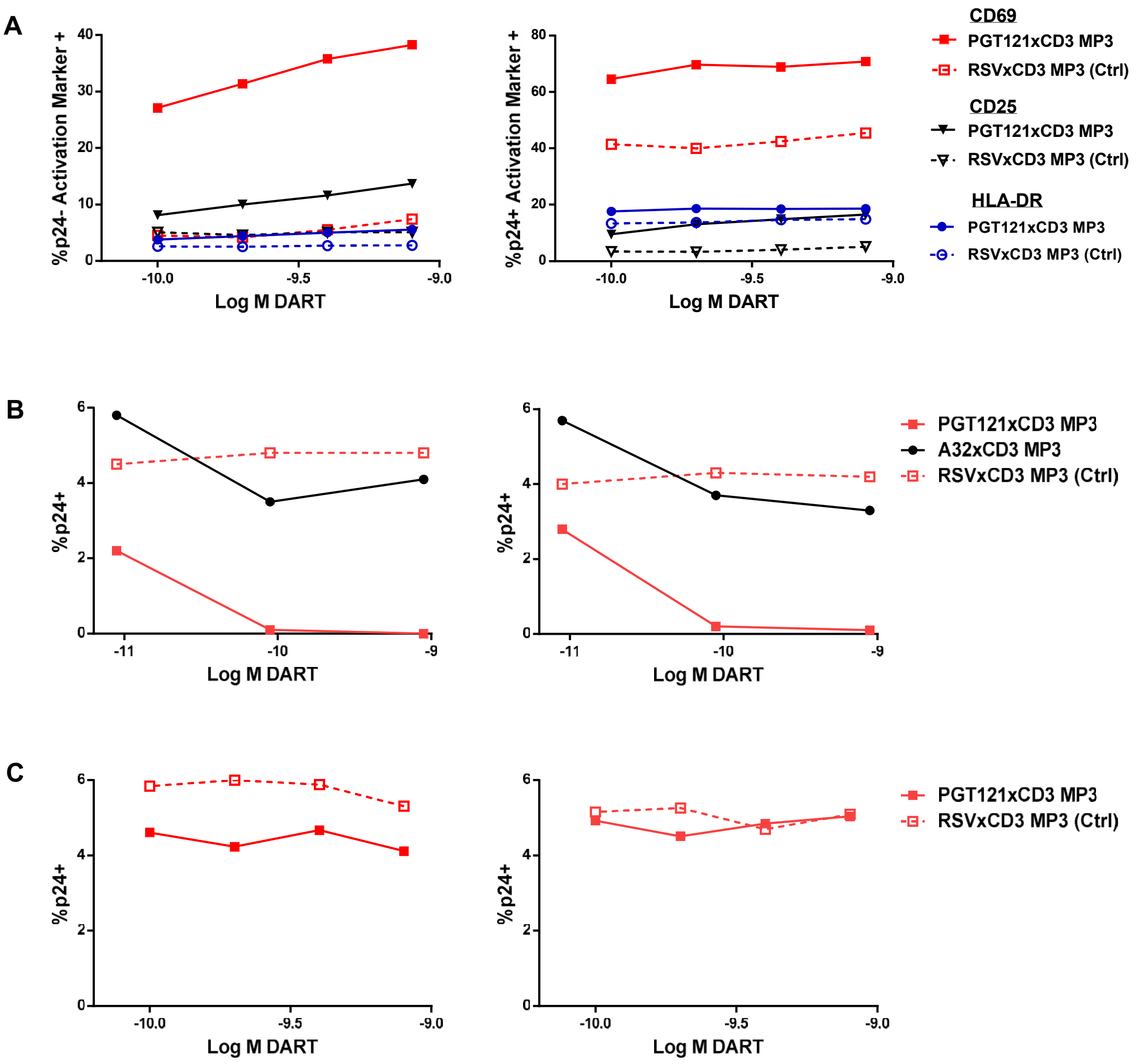

Supplementary Fig. 10. HIVxCD3 MP3 DARTs Do Not Increase HIV Spread During Productive Infection. (A) CD4 T cell Activation. PGT121xCD3 MP3 or RSVxCD3 MP3 DARTs were added to unstimulated primary CD4 T cells 24 hours post spinfection with HIV BaL. After 5 days of culture, expression of the cell surface activation markers CD25, CD69, and HLA-DR were measured on the uninfected (p24-) and the HIV-infected (p24+) fractions of CD4 T cells by FACS. **(B) Activated HIV Infection Model.** Primary CD4 T cells were activated by PMA plus ionomycin, infected with HIV (BaL) and incubated in the presence of HIVxCD3 or control MP3 DARTs. A32xCD3 MP3, derived from a non-neutralizing antibody, did not enhance HIV infection relative to RSVxCD3 MP3, while PGT121xCD3 MP3, derived from a broadly neutralizing antibody reduced the HIV infection. **(C) Resting HIV Infection Model.** Unstimulated primary CD4 T cells were infected with HIV (BaL) and incubated in the presence of HIVxCD3 or control MP3 DARTs. PGT121xCD3 MP3 did not enhance HIV infection relative to RSVxCD3 MP3.
